# Plasma proteomics mediate the association between degenerative joint diseases and dementia risk

**DOI:** 10.1016/j.jare.2025.12.044

**Published:** 2025-12-28

**Authors:** Zijian Kang, Jianzheng Zhang, Chen Zhu, Edward CH Lau, Ying Zhu, Ping Li, Kai Li, Qiang Tong, Sheng-Ming Dai

**Affiliations:** a Department of Rheumatology and Immunology, Shanghai Sixth People’s Hospital Affiliated to Shanghai Jiao Tong University School of Medicine, Shanghai, China; b Senior Department of Orthopedics, The Forth Medical Center of Chinese PLA General Hospital, Beijing, China; c College of Economics and Management, China Agricultural University, Beijing, China; d Division of Rheumatology and Clinical Immunology, Department of Medicine, Queen Mary Hospital, University of Hong Kong, Hong Kong, China; e Senior Department of Respiratory and Critical Care Medicine, The Eighth Medical Center of Chinese PLA General Hospital, Beijing, China; f College of Biological Science and Medical Engineering, Donghua University, Shanghai, China

**Keywords:** Degenerative joint disease, Osteoarthritis, Intervertebral disc degeneration, Degenerative spinal diseases, dementia, Vascular dementia, Proteomics

## Abstract

**Objective:**

To assess the association between degenerative joint diseases (DJD) and dementia risk and to identify potential proteomic mediators.

**Methods:**

We conducted a prospective cohort study using data from 500,805 participants in the UK Biobank. Three DJD diagnoses (osteoarthritis [OA], intervertebral disc degeneration [IVDD], and degenerative spinal diseases [DSD]) were identified from the clinical records. Dementia outcomes (all-cause dementia [ACD], Alzheimer’s disease [AD], and vascular dementia [VaD]) were tracked over 14.45 years. Multivariable Cox regression models were used to assess DJD-dementia associations, adjusted for demographics, lifestyle, and comorbidities. Propensity score matching (PSM) was employed for validation. Plasma proteomic profiling of 2,923 proteins was performed to identify potential mediators, followed by causal mediation analysis.

**Results:**

After a median follow-up of 14.45 years, 9,884 participants developed ACD, including 4,404 with AD and 2,224 with VaD. After adjusting for covariates, the DJD group exhibited a significantly elevated risk of ACD (HR: 1.271, 95 % CI: 1.173–1.376) and VaD (HR: 1.587, 95 % CI: 1.323–1.903), but not AD (HR: 1.107, 95 % CI: 0.982–1.248). Subgroup analyses revealed significant effect modifications according to age, activity levels, and surgical procedures. PSM analyses confirmed the robustness of these associations. Proteomic analysis identified plasma proteins associated with both DJDs and ACD risk. Mediation analysis revealed that 18 proteins, including HAVCR1 (mediation proportion 52.6 %, 95 % CI: 39.1 %–78 %), GDF15 (22 %, 95 % CI: 14.5 %–46.8 %), and COL6A3 (14.3 %, 95 % CI: 7.7 %–28.4 %), significantly mediated the association between DJDs and ACD.

**Conclusion:**

DJDs are independently associated with an increased risk of developing dementia. This correlation is mediated by systemic proteomic alterations. Our findings highlight inflammation and vascular dysfunction as central mechanisms, offering insights into risk stratification and therapeutic targets for preventing dementia.

## Highlights

• Degenerative joint diseases (DJD) are independently associated with increased risk of all-cause and vascular dementia over long-term follow-up.
• Large-scale plasma proteomic profiling identified 18 key proteins mediating the link between joint degeneration and dementia, including HAVCR1 and GDF15.
• Systemic inflammation and vascular dysfunction emerged as central biological pathways underlying the DJD–dementia association, offering targets for prevention.

## Graphical abstract

**Study overview.** In step 1, we included 406,760 non-DJD controls and 94,045 patients with DJDs who were not diagnosed with dementia at baseline. During a median follow-up of 14.45 years, 9,884 participants were diagnosed with ACD, 4,404 participants were diagnosed with AD, and 2,224 participants were diagnosed with VaD. In step 2, we utilized multivariable Cox regression analysis to adjust for covariates and investigated the relationship between DJDs and ACD risk. Additionally, several sensitivity analyses were performed to validate the robustness of these results. In Step 3, we further analyzed the association between DJDs and the ACD risk using PSM. In step 4, we performed multiple linear regression analysis to identify proteins associated with DJDs, utilized Cox regression analysis to determine proteins related to ACD, and applied mediation analysis to explore the mediating proteins that might underlie the risk of DJDs and ACD.

**Figure:**
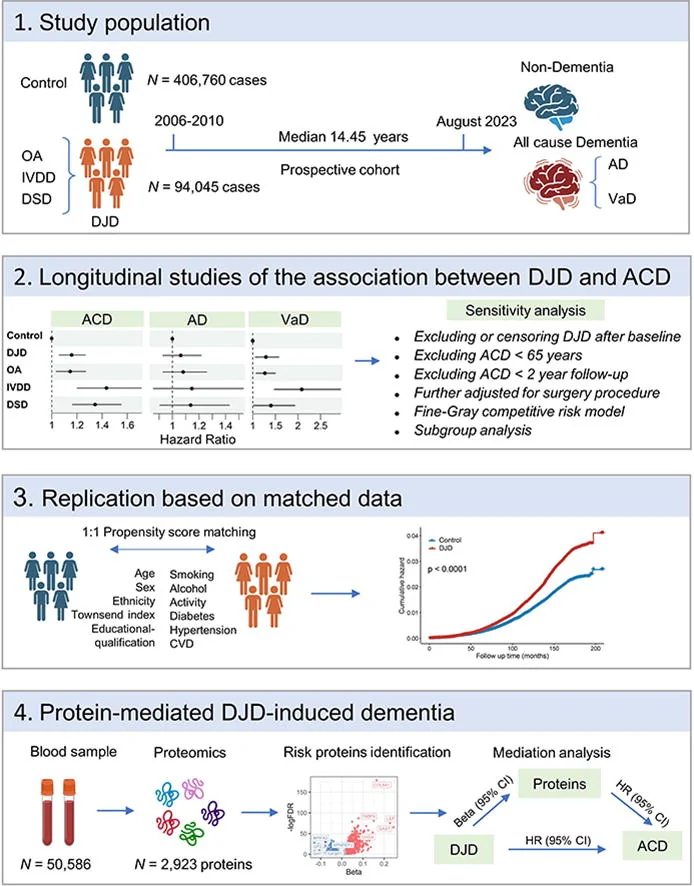

## Introduction

Degenerative joint diseases (DJDs), including osteoarthritis (OA), intervertebral disc degeneration (IVDD), and degenerative spinal diseases (DSD), are among the most prevalent causes of chronic pain and disability worldwide. DJDs significantly impact the quality of life of patients, causing chronic pain, disability, and reduced mobility. With aging populations and sedentary lifestyles, the global prevalence and incidence of DJDs are rapidly increasing, posing a significant burden on healthcare systems and society. According to the Global Burden of Disease Study, OA alone affected 595 million individuals (7.6 % of the population) in 2020, with a significant increase of 132.2 % in total cases compared with that in 1990, and is projected to rise further by 2050 due to population growth and aging [1]. Similarly, IVDD and DSD exhibit prevalence patterns with comparable age-dependent increases, further exacerbating the overall burden of DJDs [2].

DJDs are increasingly recognized not merely as localized musculoskeletal disorders but as systemic diseases with far-reaching consequences [3,4]. While biomechanical stress remains a fundamental initiating factor, chronic low-grade inflammation and metabolic dysregulation are now considered central drivers of disease progression and extra-articular complications [5]. Importantly, inflammatory mediators released from the affected joints can enter systemic circulation, contributing to systemic inflammation that affects multiple organ systems. This persistent inflammation also accelerates endothelial dysfunction and promotes atherosclerosis, thereby significantly increasing the cardiovascular risk in patients with DJDs [6]. Moreover, metabolic comorbidities, such as type 2 diabetes and metabolic syndrome, are highly prevalent in individuals with DJDs [7,8]. These metabolic disturbances not only share common inflammatory pathways with joint degeneration but also exacerbate joint pathology through oxidative stress and adipokine imbalance [9]. These associations underscore the systemic nature of DJDs and highlight the need to investigate their effects on other organ systems.

Recent studies have reported the potential association between inflammatory joint diseases and neurodegenerative diseases, particularly dementia. Studies have reported that rheumatoid arthritis (RA) and ankylosing spondylitis (AS) are associated with an increased risk of Alzheimer’s disease (AD), the most common form of dementia [[10], [11], [12]]. Proposed mechanisms include systemic inflammation, vascular dysfunction, and immune-mediated neurodegeneration [13,14]. However, significant gaps remain in our understanding of the relationship between DJDs and dementia. There is also a paucity of large-scale, comprehensive research investigating the broader spectrum of DJDs and their associations with various dementia subtypes, including vascular dementia (VaD), and all-cause dementia (ACD). Furthermore, the underlying biological mechanisms linking DJDs and dementia remain poorly understood, and the specific proteins and molecular pathways involved have not been fully elucidated.

Proteomics has recently emerged as a powerful tool for exploring the molecular mechanisms underlying disease development and comorbidity [15,16]. High-throughput proteomic platforms enable the quantification of thousands of circulating proteins, offering opportunities to identify biological mediators in complex conditions [17]. However, the application of proteomics in elucidating the potential link between DJDs and dementia remains unexplored. To address this gap, the present study leveraged multidimensional data from the UK Biobank to systematically investigate the association between DJDs and incident dementia. Through comprehensive analyses incorporating clinical records, proteomic profiles, and neuroimaging data, we aimed to elucidate the biological pathways connecting DJDs and cognitive decline, thereby providing new insights into early risk assessment and preventive interventions.

## Methods

### Data sources and study population

Analytical data were obtained from the UK Biobank (https://www.ukbiobank.ac.uk), a population-based prospective health research initiative launched during the 2006–2010 recruitment phase. This national resource comprises over half a million volunteers (aged 37–73 years) recruited through 22 assessment centers across England, providing extensive baseline characteristics through standardized protocols. Baseline evaluations included self-reported health questionnaires, physical examinations, and the collection of biospecimens. Longitudinal follow-up data were obtained through linkages to national health records and disease registries. The UK Biobank received ethical approval as a Research Tissue Bank (RTB) from the North West Multi-Centre Research Ethics Committee (MREC) (reference number: 11/NW/0382). This study used the UKB Resource under the approved application number 106397.

### Study design

This study employed a multistage analytical framework to characterize the association between DJDs and dementia. First, we included non-DJD controls and patients with DJD who were free of dementia at baseline. During the follow-up period, a subset of participants was diagnosed with incident ACD, AD, or VaD (Supplement Fig. S1). We then used multivariable Cox regression analysis to adjust for covariates and investigate the relationship between DJDs and dementia risk. Sensitivity analyses were performed to validate the robustness of these results. We then validated our results through replication analyses using propensity score matching (PSM) to ensure the consistency of the associations in the matched datasets. Finally, we performed multiple linear regression analysis to identify proteins associated with DJDs, Cox regression analysis to identify proteins related to ACD, and mediation analysis to explore the mediating proteins that might underlie the risk of DJDs and ACD.

### Assessment of DJD exposure

Diagnosis of DJD was defined using the first-occurrence data from the UK Biobank. A participant was classified as having DJD if any of the linked health-record sources, including self-reported medical conditions, hospital inpatient ICD-10 codes, and primary care Read codes, contained a relevant diagnostic code (Supplement Fig. S2). The standardized ICD-10 codes and classification criteria for DJD are documented in Supplement Table S1 [18]. Individuals with baseline DJD diagnoses were designated as the exposure group, whereas those without DJD diagnoses at baseline served as the control group. The incident DJD cases after baseline were treated as time-varying exposures.

### Assessment of covariates

To account for potential confounding factors, we incorporated a range of covariates, including demographic characteristics, socioeconomic status, lifestyle factors, and comorbidities. Regarding demographic elements, we considered age, sex (male or female), and ethnicity (White or other). Socioeconomic status was determined using the Townsend deprivation index, which incorporates four indicators of material deprivation: non-home ownership, non-car ownership, unemployment, and household overcrowding. Educational qualifications were classified based on the participants’ highest level of educational attainment. Smoking status was classified as never, previous, or current. Alcohol consumption was classified as never, occasional​ (less than twice a week), or frequent​ (at least three times a week). The physical activity level was gauged by calculating the total metabolic equivalent task (MET) minutes of all exercises performed in the preceding week and was partitioned into three categories: no activity, less than 600 MET minutes/week, 600–3000 MET minutes/week, and ≥ 3000 MET minutes/week. Comorbidities were verified using a composite approach that included self-reported medical history, examination of medication records, and inpatient diagnostic reports ​​of hypertension, diabetes, and cardiovascular diseases (CVD).

### Outcome definitions

Dementia outcomes were identified through first-occurrence data in the UK Biobank, including self-reported medical conditions, hospital inpatient ICD-10 codes, primary care Read codes, and death registry data (Supplement Fig. S2). Three primary endpoints were operationalized: (1) ACD, (2) AD, and (3) VaD. The ICD-10 Codes used for dementia diagnosis and classification in the UK Biobank [19] are shown in Supplement Table S1. Case determination also incorporates algorithmically defined outcomes, consolidated coded information from baseline assessments, and linked data sources via specialized algorithms to ensure a high positive predictive value, thereby enhancing diagnostic accuracy and reliability [19]. Longitudinal follow-up began at baseline assessments and continued until August 2023.

### Blood proteomics

Baseline blood samples were collected in ethylenediaminetetraacetic acid-coated tubes. Plasma was obtained by centrifugation, after which the supernatant was aliquoted and stored at −80 °C. Subsequently, the samples were transported to Sweden for Olink Explore Proximity Extension Assay (PEA) analysis. Antibodies conjugated to unique oligonucleotides were used for analysis. The proteomic analysis was a collaborative effort involving 13 biopharmaceutical companies and was performed using plasma samples. To minimize bias, the experimenters remained blinded to the sample characteristics and associated clinical data throughout the study. A total of 50,586 UK Biobank participants with complete proteomic data were included in the final analytical cohort.

Following stringent protein data processing steps and quality control (QC) procedures, 2,923 specific proteins were selected for further analysis. These proteins predominantly belong to the cardiometabolic, inflammatory, oncological, and neurological categories. The protein expression levels were quantified and reported as normalized protein expression (NPX) values. The NPX values were calculated by dividing the counts of each sample and detection method by those of the extended control, followed by logarithmic transformation. Variability across samples was adjusted using the median-normalized counts of the extended control, median NPX value of the specific batch, and median NPX value of the test for each batch.

### Statistical analysis

Quantitative baseline measures were characterized using the central tendency and dispersion. Normally distributed variables are presented as mean ± standard deviation, and skewed distributions are presented as median (interquartile range). Categorical parameters were summarized using frequency distributions (counts with percentages).

Cox proportional hazards models adjusted for multiple covariates were used to assess the association between DJD and dementia, with hazard ratios (HRs) and 95 % confidence intervals (CIs) serving as the primary effect estimates. A hierarchical adjustment strategy was employed: Model 1 was minimally adjusted for age, sex, and ethnicity; Model 2 was further adjusted for educational qualification, smoking status, alcohol consumption, and physical activity. Model 3 was further adjusted for hypertension, diabetes, and CVD. The proportional hazards assumption model was assessed using the Schoenfeld residuals method, and no violations were detected (Supplement Table S2).

Seven sensitivity analyses were conducted to verify the robustness of the results. First, to address the potential influence of incident DJD cases after the baseline, participants who developed DJD during follow-up were either excluded or censored at the date of diagnosis. Multivariable Cox proportional hazards models were used to evaluate the association between DJD status and the risk of ACD. Second, to minimize potential reverse causality bias, participants who developed dementia within two years of the baseline were excluded, and the primary analyses were repeated. Third, considering the low prevalence of dementia in individuals before age 65 years, participants aged < 65 years at baseline were excluded, and primary analyses were replicated [10]. Fourth, to address potential outcomes from mortality events, we implemented multivariable-adjusted competing risk regression models using Fine and Gray’s subdistribution hazard approach. Fifth, to address the potential influence of major joint or spinal surgeries, Model3 were further adjusted for surgical procedures, including knee or hip replacement and lumbar or spinal surgeries. Sixth, subgroup analyses were conducted to evaluate whether the relevant covariates modified the association between DJDs and dementia. Interaction terms for age, sex, ethnicity, Townsend deprivation index, smoking status, alcohol consumption, physical activity level, comorbidities, and surgical procedure were incorporated to assess the stratified effects across the subgroups. Seventh, PSM was implemented to minimize confounding factors [20]. We estimated propensity scores for DJD exposure based on the baseline covariates. Model 1 was matched for age, sex, and ethnicity. Model 2 was further matched for educational qualification, Townsend deprivation index, smoking status, alcohol consumption, and physical activity. Model 3 was matched for all covariates from Model 2, plus hypertension, diabetes, and CVD. A 1:1 nearest-neighbor matching algorithm with caliper widths ≤ 0.1 standard deviation of the propensity score logit ensured covariate balance. Post-matching equilibrium was verified using standardized mean differences (SMDs) of < 10 % across all covariates. We then performed a stratified Cox proportional hazards model using the match ID generated during the PSM process as the stratification variable to account for within-pair dependency.

Based on previous analyses, plasma proteomics has been identified as potential mediators [16,21]. To handle potential biases from missing covariate data, we first applied multivariate imputation using the multivariate median imputation. Multiple linear regression models were used to examine the association between DJDs and these proteins. Cox regression models were used to investigate the relationship between biomarkers and the occurrence of dementia. The dose–response relationship between protein expression levels and ACD risk was examined using restricted cubic spline (RCS) regression. The RCS model incorporated four knots positioned at the 5th, 35th, 65th, and 95th percentiles of protein expression distribution.

To gain deeper biological insights, we compared the significant proteins with the complete Olink protein set as a background reference. Fisher’s exact test was used to determine statistical significance, with p-values adjusted for multiple comparisons using the Benjamini-Hochberg procedure to control the false-discovery rate (FDR). Proportion-mediated (PM) analysis was used to evaluate the mediating effect of representative proteins on the association between DJDs and dementia risk using the mediation package. The 95 % CIs for PM were computed using nonparametric bootstrapping with 1000 iterations.

All the analyses were conducted using R software (version 4.2.2). An FDR-adjusted p-value < 0.05 was considered statistically significant for proteomic analyses. A two-tailed P-value < 0.05 was deemed statistically significant for the prospective association analysis.

## Results

### Participant Selection and Baseline Characteristics

To investigate the relationship between DJDs and the risk of developing dementia and to identify circulating proteomic mediators that may underlie this association, we utilized a multistage analytical framework. The UK Biobank cohort initially included 502,357 participants. After excluding 1,297 participants lost to follow-up and 255 participants with baseline dementia (Supplement Fig. S1), we included three main DJDs that largely influence people’s health, [18,22] including OA, IVDD, and DSD at baseline as the exposure group and those without DJD as the control group. Finally, our cohort comprised 500,805 participants, including 406,760 non-DJD controls and 94,045 patients with DJD.

Significant baseline differences were observed between the two groups (Table 1). Patients with DJDs had a higher mean age (59.65 vs. 55.81 years) and a higher percentage of females (59.0 % vs. 53.3 %) than those without DJDs. The DJD group also included more Caucasian individuals (95.5 % vs. 93.6 %) and had a higher prevalence of obesity (33.2 % vs. 22.3 %) than that of the non-DJD group. Regarding socioeconomic status, a slightly higher proportion of patients with DJDs were in the most deprived category (26.3 % vs. 24.6 %). In terms of lifestyle, patients with DJDs were more likely to smoke (49.3 % vs. 43.9 %). The educational level was slightly lower in the DJD group, with fewer individuals holding college or university degrees (8.5 % vs. 11.9 %). Additionally, hypertension, diabetes, and CVD were more common in the DJD group (hypertension, 58.9 % vs. 49.3 %; diabetes, 4.8 % vs. 3.6 %; CVD: 7.3 % vs. 4.1 %). The baseline characteristics of the three DJDs are presented in Table 1**.** These differences established distinct profiles for the cohorts at baseline.

**Table 1:** Baseline characteristics of the study population.

**Variable**	**Control**	**DJD**		**OA**		**IVDD**		**DSD**	
	**N = 406,760**	**N = 94,045**	**P value**	**N = 64,482**	**P value**	**N = 17,401**	**P value**	**N = 27,841**	**P value**
**Age (mean (SD))**	55.81 (8.16)	59.65 (7.01)	<0.001	60.65 (6.39)	<0.001	57.30 (7.80)	<0.001	59.37 (7.15)	<0.001
**Sex (%)**			<0.001		<0.001		<0.001		<0.001
Female	216,986 (53.3)	55,509 (59.0)		39,278 (60.9)		9024 (51.9)		16,942 (60.9)	
Male	189,774 (46.7)	38,536 (41.0)		25,204 (39.1)		8377 (48.1)		10,899 (39.1)	
**Ethnicity (%)**			<0.001		<0.001		<0.001		<0.001
White	380,741 (93.6)	89,854 (95.5)		61,714 (95.7)		16,579 (95.3)		26,598 (95.5)	
Non-white	23,222 (5.7)	3549 (3.8)		2303 (3.6)		723 (4.2)		1074 (3.9)	
**BMI (%)**			<0.001		<0.001		<0.001		<0.001
Underweight	2299 (0.6)	323 (0.3)		178 (0.3)		57 (0.3)		138 (0.5)	
Normal	139,311 (34.2)	22,532 (24.0)		13,997 (21.7)		4558 (26.2)		7158 (25.7)	
Overweight	172,147 (42.3)	39,311 (41.8)		26,364 (40.9)		7593 (43.6)		11,802 (42.4)	
Obesity	90,601 (22.3)	31,219 (33.2)		23,502 (36.4)		5064 (29.1)		8512 (30.6)	
**Townsend index (%)**			<0.001		<0.001		0.878		<0.001
1 (least deprived)	102,955 (25.3)	22,343 (23.8)		15,019 (23.3)		4357 (25.0)		6382 (22.9)	
2	101,652 (25.0)	23,270 (24.7)		15,778 (24.5)		4401 (25.3)		6773 (24.3)	
3	101,653 (25.0)	23,607 (25.1)		16,231 (25.2)		4354 (25.0)		7048 (25.3)	
4 (most deprived)	99,992 (24.6)	24,711 (26.3)		17,381 (27.0)		4266 (24.5)		7600 (27.3)	
**Drinking (%)**			<0.001		<0.001		<0.001		<0.001
Never	30,985 (7.6)	9473 (10.1)		6726 (10.4)		1654 (9.5)		3118 (11.2)	
Occasional	195,399 (48.0)	46,984 (50.0)		32,440 (50.3)		8510 (48.9)		14,293 (51.3)	
Frequent	179,164 (44.0)	37,315 (39.7)		25,123 (39.0)		7196 (41.4)		10,359 (37.2)	
**Smoking (%)**			<0.001		<0.001		<0.001		<0.001
Never	225,602 (55.5)	47,067 (50.0)		32,062 (49.7)		8631 (49.6)		13,630 (49.0)	
Previous	136,034 (33.4)	36,391 (38.7)		25,645 (39.8)		6492 (37.3)		10,648 (38.2)	
Current	42,823 (10.5)	9963 (10.6)		6332 (9.8)		2174 (12.5)		3368 (12.1)	
**Activity level (%)**			<0.001		<0.001		<0.001		<0.001
Never	5739 (1.4)	2354 (2.5)		1632 (2.5)		537 (3.1)		872 (3.1)	
Low	51,306 (12.6)	11,334 (12.1)		7569 (11.7)		2338 (13.4)		3495 (12.6)	
Medium	162,006 (39.8)	32,476 (34.5)		21,764 (33.8)		6239 (35.9)		9345 (33.6)	
High	96,734 (23.8)	22,017 (23.4)		15,033 (23.3)		4073 (23.4)		6305 (22.6)	
**Educational Qualification (%)**			<0.001		<0.001		<0.001		<0.001
College or University	48,500 (11.9)	8023 (8.5)		5127 (8.0)		1805 (10.4)		2156 (7.7)	
A levels/AS levels	12,204 (3.0)	2326 (2.5)		1517 (2.4)		488 (2.8)		679 (2.4)	
O levels/GCSEs	104,401 (25.7)	21,022 (22.4)		14,058 (21.8)		4032 (23.2)		6215 (22.3)	
CSEs or equivalent	57,720 (14.2)	12,649 (13.4)		8402 (13.0)		2450 (14.1)		3777 (13.6)	
NVQ or HND or HNC	114,251 (28.1)	24,562 (26.1)		16,570 (25.7)		4970 (28.6)		6788 (24.4)	
**Comorbidities (%)**									
Hypertension	200,403 (49.3)	55,378 (58.9)	<0.001	39,642 (61.5)	<0.001	9382 (53.9)	<0.001	16,112 (57.9)	<0.001
Diabetes	14,563 (3.6)	4500 (4.8)	<0.001	3316 (5.1)	<0.001	741 (4.3)	<0.001	1302 (4.7)	<0.001
CVD	16,586 (4.1)	6829 (7.3)	<0.001	5013 (7.8)	<0.001	1118 (6.4)	<0.001	2351 (8.4)	<0.001

**Abbreviations:** DJD: Degenerative joint disease; OA: Osteoarthritis; IVDD: Intervertebral disc degeneration; DSD: Degenerative spinal diseases; BMI: body mass index; A levels/AS levels: Advanced levels/Advanced Subsidiary levels; O levels/GCSEs: Ordinary levels/General Certificate of Secondary Education; CSEs or equivalent: Certificate of Secondary Education or equivalent; NVQ or HND or HNC: National Vocational Qualification or Higher National Diploma or Higher National Certificate; CVD: Cardiovascular diseases.

### Djds increase the risk of developing dementia

We designed a cohort study to evaluate the effects of DJDs on the risk of developing ACD, AD, and VaD. After a median follow-up of 14.45 years, 9,884 participants were diagnosed with ACD, including 4,404 participants were diagnosed with AD, and 2,224 participants were diagnosed with VaD. We conducted stepwise-adjusted Cox proportional hazards regression analyses adjusted for multiple covariates, including age, sex, ethnicity, educational qualification, Townsend deprivation index, smoking status, alcohol consumption, physical activity level, hypertension, diabetes, and CVD.

In the fully adjusted model, the DJD group exhibited an elevated risk of ACD compared to the control group, with an HR of 1.271 (95 % CI: 1.173–1.376, Fig. 1A**,** Supplement Table S3). Stratified analyses according to specific DJD subtypes showed consistently elevated risks: OA (HR: 1.177, 95 % CI: 1.079–1.285), IVDD (HR: 1.488, 95 % CI: 1.279–1.730), and DSD (HR: 1.569, 95 % CI: 1.400–1.760).

**Fig. 1:**
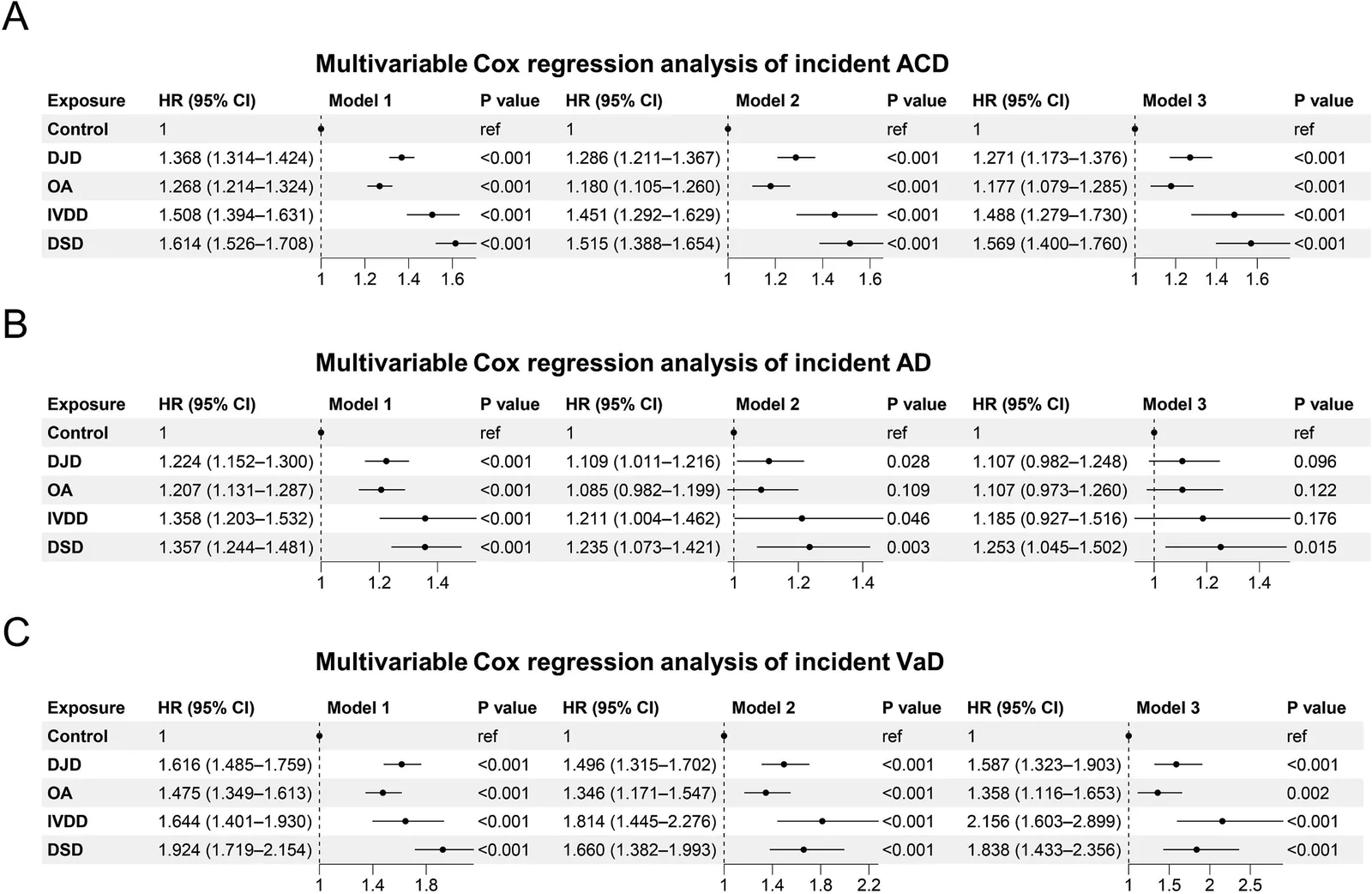
**Associations between DJDs and dementia risk estimated using multivariable-adjusted Cox proportional hazard models. (A-C)**. Forest plots showing the HRs for ACD (A), AD (B), and VaD (C) risk associated with DJDs. Three models were fitted: Model 1 adjusted for baseline demographics (age, sex and ethnicity); Model 2 additionally adjusted for ethnicity, educational qualification, Townsend index, smoking status, alcohol consumption and physical activity; and Model 3 further adjusted for comorbidities (hypertension, diabetes, and CVD). The incident DJD cases after baseline were treated as a time-varying exposure. **Abbreviations** CVD: Cardiovascular diseases.

Further analysis focusing on dementia subtypes revealed that the DJD group had a significantly increased risk of VaD, with an HR of 1.587 (95 % CI: 1.323–1.903). Among the DJD subtypes, OA was associated with an HR of 1.358 (95 % CI: 1.116–1.653), IVDD with an HR of 2.156 (95 % CI: 1.603–2.899), and DSD with an HR of 1.838 (95 % CI: 1.433–2.356) for VaD (Fig. 1**B and C**, Supplement Table S4 and S5). In contrast, no statistically significant association was observed between DJD and AD, with an overall HR of 1.107 (95 % CI: 0.982–1.248) compared to the control group (Fig. 1**B and C**, Supplement Tables S4 and S5).

To verify the robustness of our findings, we conducted a series of sensitivity analyses. Considering the potential influence of incident DJD cases occurring after the baseline, participants who developed DJD during follow-up were either excluded or censored at the date of diagnosis. Cox regression analysis indicated that the overall DJD group, as well as its subgroups, were associated with an increased risk of ACD and VaD (Supplement Tables S6-S9). To account for the potential confounding effect of the surgical procedure, we further adjusted surgery based on Model 3. After this adjustment, DJD and its subgroups remained significantly associated with a higher risk of ACD (Supplement Table S10). To minimize the potential for reverse causality and exclude early onset dementia cases, individuals with less than two years of follow-up or those diagnosed with ACD before the age of 65 years were excluded. After these exclusions, the association between total DJDs and the increased risk of ACD remained significant. Among the specific DJD types, OA, IVDD, and DSD also demonstrated significant associations (Supplement Tables S11 and S12**)**. Considering the competing risk of death, we employed the Fine-Gray method. DJD and its subgroups were significantly associated with the risk of dementia **(Supplement Figs. S3 and S4, Supplement Table S13)**.

Pain-stratified analyses revealed a clear gradient of dementia risk: painful DJD phenotypes displayed a substantially higher ACD risk (HR: 1.597, 95 %CI: 1.432–1.782) than non-painful DJD patients, whose associations were weaker (HR: 1.315, 95 %CI: 1.142–1.513, **Supplement Table S14**). This pattern was consistent across OA, IVDD, and DSD, suggesting that pain, reflecting greater symptomatic burden, may amplify the dementia risk associated with DJDs.

Subgroup analyses further confirmed the association between DJDs and an increased risk of ACD across various stratified populations. Notably, significant effect modifications were observed according to age, activity level, and surgery. Specifically, the risk associated with DJDs was most prominent in individuals aged < 55 years (HR: 2.254, 95 % CI: 1.773–2.865), those without regular daily activity (HR: 1.557, 95 % CI: 1.036–2.339), and those who had undergone surgery (HR: 2.165, 95 % CI: 1.580–2.967), compared to their corresponding reference groups without DJDs (**Supplement Figs. S5–S8)**. Joint effect analyses of DJD status with physical activity and surgical procedures indicated that individuals with DJD, OA, or DSD who lacked regular physical activity had a higher risk of ACD compared with those who engaged in regular activity. Likewise, individuals with DJD or OA who had undergone surgical procedures exhibited a greater risk of developing ACD than those without surgical history (**Supplement Fig. S9)**.

We also employed PSM to select an equal number of controls matched for all covariates with the corresponding DJD group. The absolute SMD was < 0.1, indicating minimal differences between the matched groups (**Supplement Table S15**). When Cox proportional hazards analyses were restricted to the matched data, similar trends in the direction and magnitude of associations were observed between DJD conditions and ACD incidence, thereby validating the robustness of our results (Fig. 2A-D, **Supplement Table S16**).

**Fig. 2:**
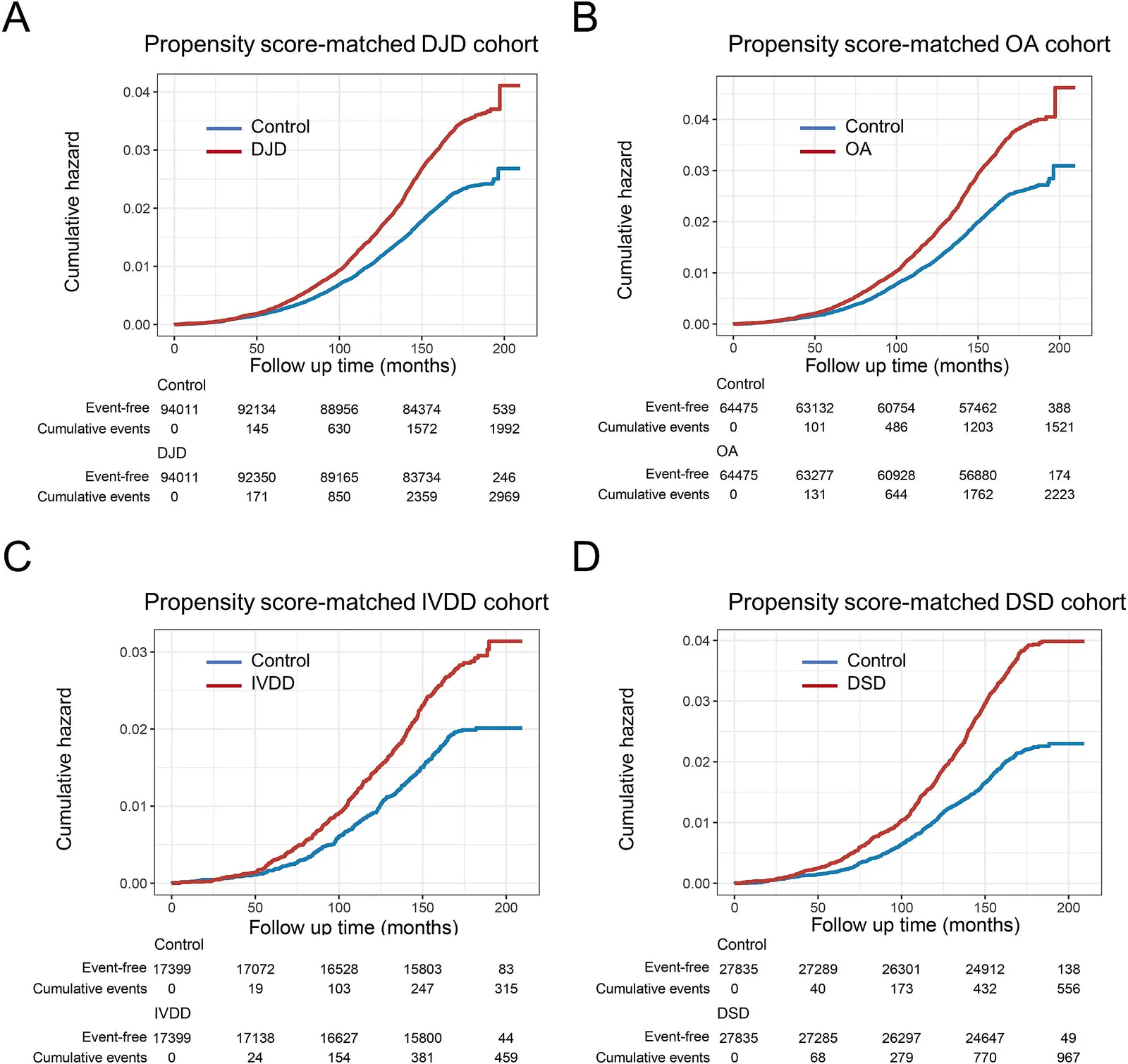
**Association between different DJD subtypes and ACD risk using PSM**. **(A-D).** The cumulative hazard curves illustrate the risk of ACD in DJD (A), OA (B), IVDD (C), and DSD (D) and the matched control group during the follow-up period. Matching was performed based on age, sex, and ethnicity, educational qualification, Townsend deprivation index, smoking status, alcohol consumption, physical activity, hypertension, diabetes, and CVD. The numbers of event-free individuals and cumulative events are provided below each plot to indicate the sample size and event occurrence at different follow-up intervals. **Abbreviations** PSM: propensity score matching.

In summary, our findings suggest that DJDs are significantly associated with an increased risk of developing dementia. The influence of DJDs on dementia risk varies according to activity levels and surgical procedures.

### Identification of proteins associated with DJDs

To elucidate the potential pathways through which DJDs may influence ACD, we integrated plasma proteomics, as proteins are potential mediators of this association. We assessed the relationship between DJDs and circulating biomarkers using a multiple linear regression model. Our findings revealed that DJDs significantly upregulated proteins such as COL9A1, GSAT, LEP, FABP4, and CHGA, while downregulating proteins such as IFGBP1, BPIFA2, and GH1 (Fig. 3A**).** Among the DJD significantly associated proteins (FDR < 0.05), the top 50 with positive β-values and the top 50 with negative β-values are presented in **Supplement Table S17**. DJD-associated proteins are implicated in immune reactions, cytokine activities, and the migration of inflammatory cells (Fig. 3B**, Supplement Table S18**).

**Fig. 3:**
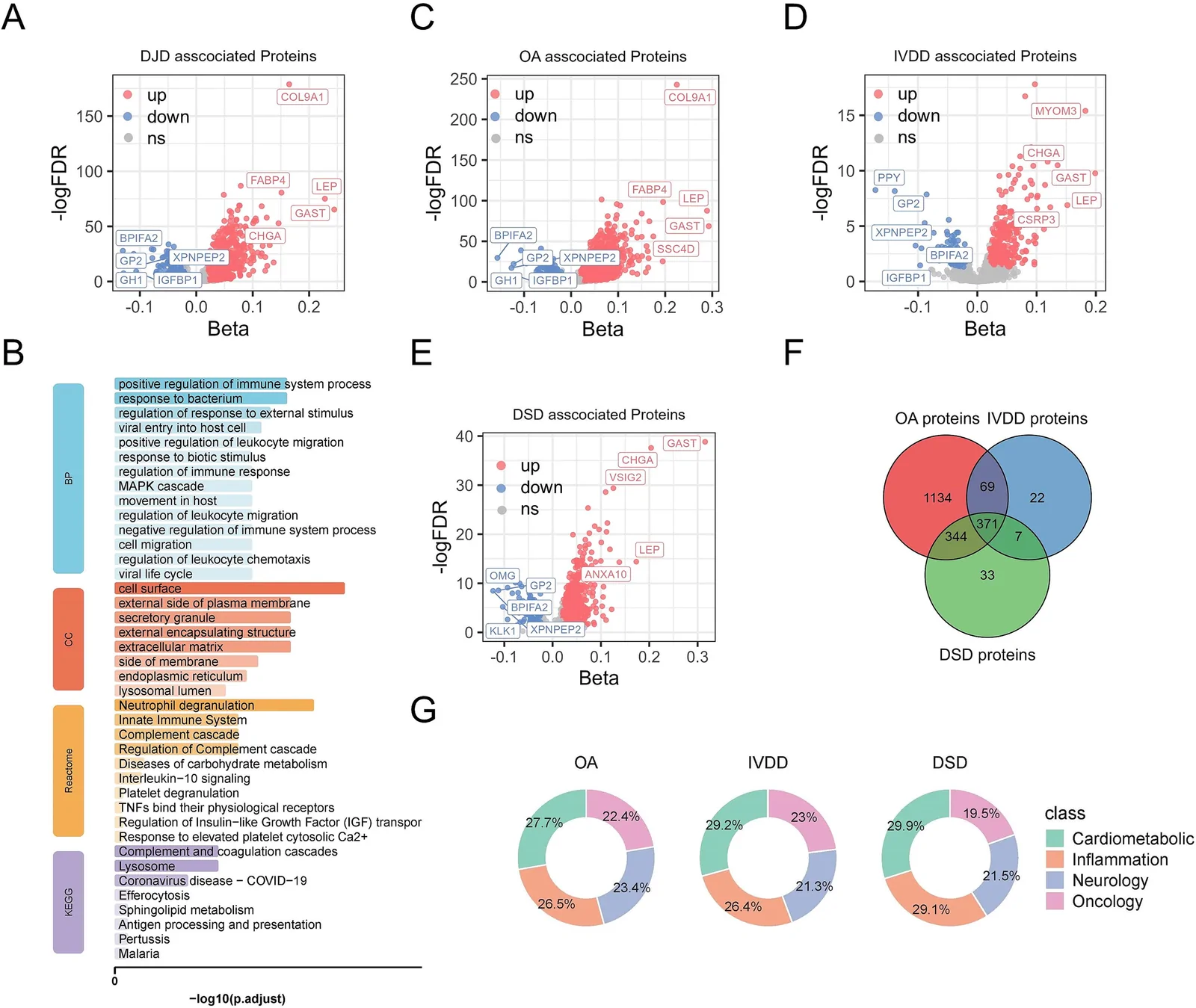
**Identification and functional enrichment of proteins associated with DJDs.** (**A).** Volcano plot showing proteins associated with DJDs, identified through multiple linear regression, with the x-axis representing beta values and the y-axis representing FDR values. The red and blue dots indicate upregulated and downregulated proteins, respectively. **(B).** Functional enrichment analysis of DJD-associated proteins categorized by BP, CC, MF, Reactome, and KEGG pathways. (**C-E).** Volcano plot showing proteins associated with OA (C), IVDD (D), and DSD (E), identified through multiple linear regression, with the x-axis representing beta values and the y-axis representing FDR values. The red and blue dots indicate upregulated and downregulated proteins, respectively. **(F).** Venn plot showing the overlap of proteins across different DJDs.**(G)**. Pie charts illustrating the distribution of protein classes (cardiometabolism, inflammation, neurology, oncology) associated with each disorder. **Abbreviations** FDR: False discovery rate; BP: Biological Process; CC: Cellular Component; MF: Molecular Function.

We further explored the protein expression profiles associated with specific DJD subtypes **(**Fig. 3**C-E,** Supplement Tables S19-S21**)**. These proteins are involved in inflammation, cardiometabolic processes, oncology, and neurology, with some proteins shared across different DJD subtypes. Our analysis identified 371 proteins, including GAST, LEP, CHGA, and FABP4, that were consistently upregulated across all DJD subtypes, indicating that these proteins are commonly influenced by multiple DJDs (Fig. 3**F-G**).

Furthermore, our investigation revealed that distinct proteins were upregulated in each DJD subtype. Specifically, the upregulation of proteins such as IL-1RN, FABP1, IL-6, and OXT was observed in patients with OA. Similarly, in IVDD, proteins such as MYOM3, CSRP3, and ADH4, exhibited increased expression. In DSD, the upregulation of CHIT1, REN, and LAMP3 proteins was also noted (**Supplement Fig. S10)**. These findings highlight the diverse protein expression patterns associated with different DJD subtypes and their potential implications for disease-specific mechanisms.

Functional enrichment analysis revealed significant enrichment in biological processes related to immune responses, inflammatory processes, cell adhesion and migration, cytokine-cytokine receptor interaction, complement and coagulation cascades, and apoptosis (**Supplement Fig. S11, Supplement Table S22**). These results suggest that proteins associated with DJDs are integral to the complex network of immune and inflammatory responses and cellular processes that may contribute to neurodegeneration.

### Identification of proteins associated with ACD

To elucidate the association between these biomarkers and ACD incidence, we employed Cox regression analysis. We identified 820 proteins significantly associated with an increased risk of ACD, including MENT, GFAP, NEFL, PLAUR, and GDF15. In contrast, proteins such as APOE, EGFR, ITGAV, and CLEC3B were associated with a reduced risk of ACD **(**Fig. 4A**)**. Among the ACD significantly associated proteins (FDR < 0.05), the top 50 with the highest HR values and the top 50 with the lowest HR values are presented in **Supplement Table S23.** ACD-associated proteins are implicated in wound healing, Rap1 signaling, cytokine-cytokine receptor interaction, MAPK signaling, and extracellular matrix organization, highlighting the involvement of neuroinflammation, cell signaling, and extracellular matrix remodeling in ACD pathogenesis (Fig. 4B**, Supplement Table S24**). Cumulative hazard plots further demonstrated that these proteins, exemplified by the six proteins with the highest HRs, were significantly associated with the risk of ACD (Fig. 4C**)**. Subsequently, we utilized the RCS method to explore the dose–response relationships between the six proteins and ACD risk. The RCS curves revealed distinct dose–response relationships between protein expression levels and ACD risk. Specifically, MENT, GFAP, and NEFL exhibited nonlinear relationships with ACD risk, whereas GDF15, PLAUR, and FSTL1 showed nonlinear dose–response associations with ACD risk (Fig. 4D**)**. These findings suggest that the association between protein expression levels and ACD risk is complex and may involve both linear and nonlinear patterns.

**Fig. 4:**
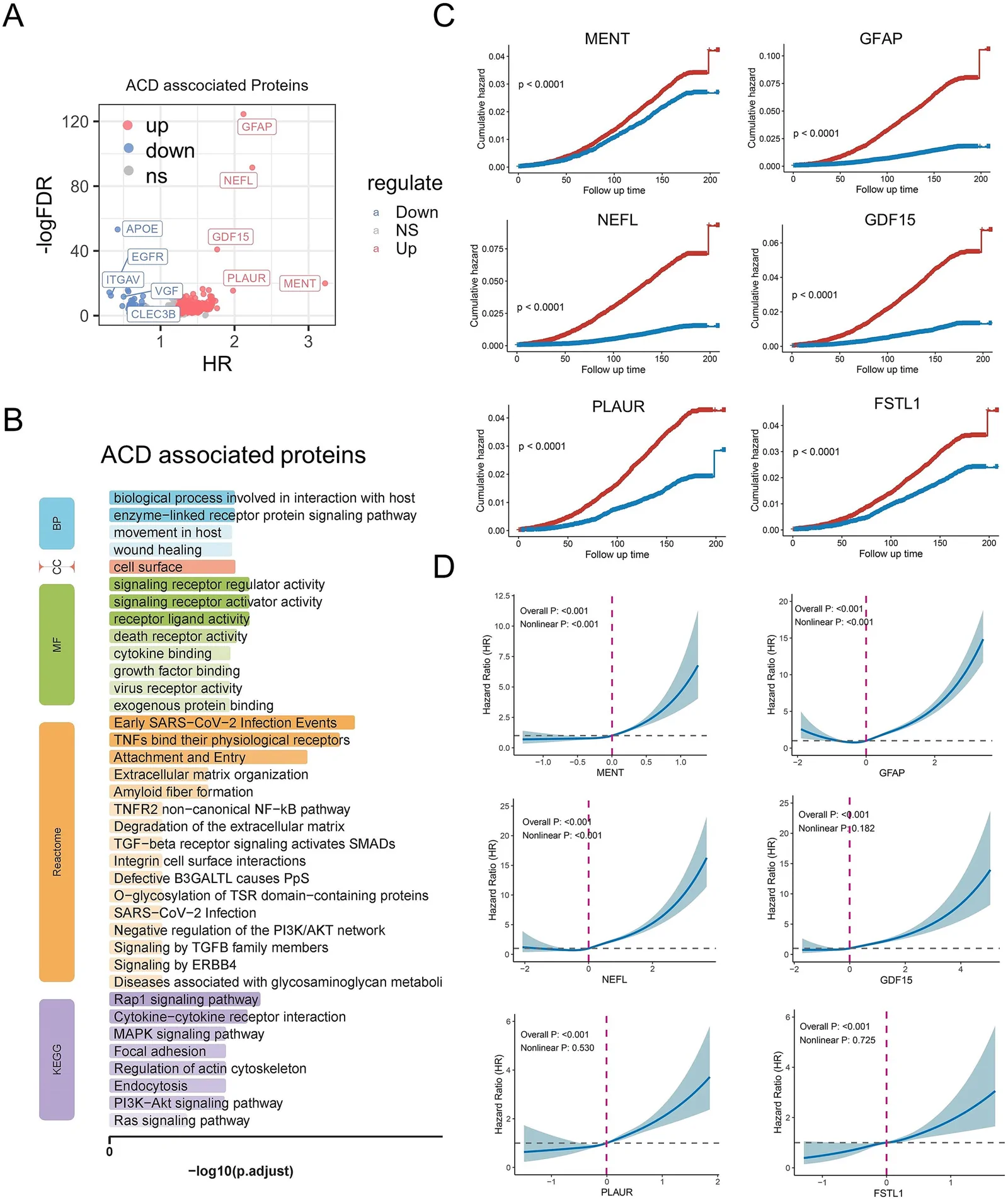
Identification and functional enrichment of proteins associated with ACD risk. **(A).** Volcano plot showing proteins associated with ACD identified using Cox regression, with the x-axis representing HR values and the y-axis representing FDR values. The red and blue dots indicate upregulated and downregulated proteins, respectively. **(B).** Functional enrichment analysis of ACD-associated proteins categorized by GO, KEGG, and Reactome. The bar charts depict the top-enriched biological processes, pathways, and molecular functions associated with ACD risk. **(C).** Cumulative hazard curves illustrating the risk of ACD according to the expression levels of top-ranked proteins during the follow-up period. The population was divided into high-expression and low-expression groups based on the median protein expression levels. The curves show the time-dependent risk stratification, with higher expression levels associated with increased risk. **(D)**. RCS curves illustrating the dose–response relationship between the expression levels of top-ranked proteins and ACD risk. The x-axis represents the protein expression levels, and the y-axis represents the HR for ACD. The curves depict the linear/nonlinear relationship between protein expression and ACD risk, with confidence intervals shown as shaded areas. **Abbreviations** RCS**:** Restricted cubic spline.

### Mediation analysis of proteins in the relationship between DJDs and ACD

To further elucidate the potential mechanisms linking DJDs to ACD, we performed a mediation analysis focusing on these proteins. By integrating DJD-associated proteins (FDR < 0.05), ACD-associated proteins (FDR < 0.05), and candidate mediators (FDR < 0.05, PM > 1 %), we identified a total of 18 proteins that were shared across all three screening approaches and significantly mediated the DJD-ACD association (Table 2**)**.

**Table 2:** Mediation analysis of proteins in the association between DJDs and ACD risk.

**Proteins**	**Beta (95 % CI)**	**FDR (Glm)**	**HR (95 % CI)**	**FDR (Cox)**	**Mediation proportion (95 % CI), %**	**FDR (Mediation)**
HAVCR1	0.096 (0.081–0.112)	***	1.19 (1.111–1.275)	***	52.6 (39.1–78)	***
GDF15	0.087 (0.076–0.098)	***	1.765 (1.630–1.911)	***	22.0 (14.5–46.8)	***
COL6A3	0.097 (0.088–0.106)	***	1.423 (1.271–1.594)	***	14.3 (7.7–28.4)	***
IGFBP4	0.089 (0.078–0.099)	***	1.369 (1.249–1.500)	***	11.6 (7.2–29.6)	***
CHGA	0.146 (0.128–0.164)	***	1.18 (1.123–1.239)	***	10.2 (6.1–20.3)	***
MMP12	0.083 (0.070–0.097)	***	1.324 (1.223–1.433)	***	10.1 (6–21.2)	***
LGALS4	0.084 (0.071–0.098)	***	1.321 (1.223–1.427)	***	9.5 (4.9–16.8)	***
KLK4	0.082 (0.066–0.097)	***	1.299 (1.216–1.389)	***	9.4 (5.3–21)	***
VSIG2	0.101 (0.088–0.113)	***	1.24 (1.151–1.336)	***	8.8 (4.8–17.2)	***
CHI3L1	0.101 (0.082–0.119)	***	1.213 (1.151–1.279)	***	8.5 (5.4–15.3)	***
ACE2	0.105 (0.092–0.118)	***	1.206 (1.113–1.308)	***	8.0(3.3–17.7)	***
TFF1	0.091 (0.072–0.110)	***	1.188 (1.128–1.250)	***	6.8 (3.8–14.9)	***
IL6	0.118 (0.099–0.136)	***	1.141 (1.084–1.201)	***	6.6 (3.7–12.6)	***
CDCP1	0.116 (0.103–0.128)	***	1.149 (1.057–1.250)	***	6.3 (2.4–13)	***
ADGRG1	0.099 (0.082–0.116)	***	1.141 (1.086–1.198)	***	5.5 (3–11.7)	***
FABP1	0.122 (0.099–0.146)	***	1.118 (1.067–1.170)	***	5.4 (2.9–10.3)	***
AGR2	0.084 (0.061–0.108)	***	1.131 (1.080–1.184)	***	4.6 (2.2–9.0)	***
SNCG	0.081 (0.063–0.097)	***	1.118 (1.046–1.196)	***	3.6 (1.1–8.4)	***

**Abbreviations:** FDR: false-discovery rate; HR: hazard ratios; CI: confidence intervals.

For instance, HAVR1 exhibited the highest mediation proportion, accounting for 52.6 % (95 % CI: 39.1 %–78 %) of the mediation effect. GDF15 mediated 22 % (95 % CI: 14.5 %–46.8 %) and collagen protein COL6A3 mediated 14.3 % (95 % CI: 7.7 %–28.4 %) of the relationship. Other notable proteins included inflammatory proteins IL-6 (6.6 %), MMP12 (10.1 %), and CHI3L1 (8.5 %), cell adhesion proteins LGALS4 (9.5 %), ADGRG1 (5.5 %), and CDCP1 (6.3 %), growth factor regulators IGFBP4 (11.6 %), and vascular markers ACE2 (8.0 %), which exhibited significant mediation proportions (Table 2**)**.

These findings highlight the protein mediators linking DJDs to ACD and offer mechanistic insights into potential therapeutic targets for preventing or treating dementia associated with musculoskeletal conditions.

## Discussion

In this study, we investigated the association between DJDs and dementia risk using a large population-based dataset enriched with clinical diagnoses and proteomic profiles. Our analysis revealed that multiple DJDs were significantly associated with an increased risk of developing dementia. Multiple plasma proteins have been identified to mediate the association between DJD and dementia. These findings provide molecular and epidemiological evidence of a mechanistic link between systemic DJDs and dementia.

Although inflammatory joint diseases or autoimmune diseases have been linked to cognitive decline or dementia, [10,11,23] the relationship between DJDs and dementia remains unclear. Leveraging large-scale longitudinal data from the UK Biobank and proteomic validation, our study revealed novel associations that have been previously underreported. Specifically, we identified an elevated risk of dementia across distinct DJD categories, including OA, IVDD, and DSD. These findings challenge the conventional view that DJDs are primarily localized or biomechanical [24]. Furthermore, the stronger association observed between DJDs and VaD rather than AD reinforces the hypothesis that cerebrovascular and inflammatory mechanisms might be more prominent in mediating this relationship. The vascular burden, common in DJD patients, may lead to subclinical brain infarctions or white matter changes that accelerate vascular cognitive impairment [25]. Additionally, chronic low-grade inflammation, a hallmark of DJDs, has been implicated in endothelial dysfunction and blood–brain barrier breakdown, which are both pivotal in VaD pathogenesis [[26], [27], [28]].

AD is a neurodegenerative disorder primarily characterized by amyloid-beta (Aβ) deposition and tau pathology. Although vascular risk factors have been implicated in AD progression, the underlying mechanisms are generally considered to be amyloid-driven. Our study found no significant association between DJDs and AD, suggesting that the cognitive effects of DJDs may occur via amyloid-independent mechanisms. This highlights the importance of exploring alternative pathways in the pathogenesis of dementia, particularly in populations affected by chronic DJDs. This interpretation is supported by neuroimaging research showing that AD and VaD exhibit distinct patterns of brain changes. While AD is typically linked to medial temporal lobe atrophy, VaD is associated with periventricular white matter hyperintensities and lacunar infarcts, lesions that are more prevalent among individuals with systemic inflammation and vascular comorbidities [29]. Future research should focus on elucidating the specific molecular and cellular pathways linking DJDs to neurodegeneration, which may provide novel therapeutic targets for preventing or mitigating cognitive decline in these populations.

Subgroup analyses revealed significant effect modifications by age, activity level, and surgical procedure, highlighting the heterogeneity in susceptibility to DJD-related dementia. The particularly elevated risk among younger individuals (<55 years) suggests that DJDs may contribute to neurodegenerative changes through early-or midlife mechanisms independent of age-related vascular burden. The stronger association in participants without physical activity underscores the potential protective role of exercise in mitigating systemic inflammation and enhancing neurovascular health, both of which are critical in dementia pathogenesis. Although joint replacement surgery is generally intended to relieve pain and restore mobility, our findings showed that patients with OA/DJD who underwent surgery had a higher risk of ACD than those who did not. This association likely reflects confounding by indication, as patients selected for surgery typically have more advanced joint degeneration, greater pain burden, longer disease duration and prolonged systemic inflammation, which may predispose to neurodegeneration. While surgery may improve mobility, the potential cognitive benefits may have been statistically adjusted for (e.g., via physical activity), and perioperative stress or pre-existing disease burden may outweigh those benefits [[30], [31]]. As this is an observational study, causality cannot be inferred; thus, the elevated risk observed among surgically treated patients likely represents their greater underlying disease burden and neurocognitive vulnerability, rather than a direct harmful effect of surgery itself. These findings emphasize the need to consider demographic factors, physical activity levels, and surgical history when evaluating dementia risk in the DJD population and point to the potential benefit of targeted prevention strategies in subgroups that may be overlooked despite elevated vulnerability.

Proteomic analysis identified several proteins that significantly mediate the association between DJDs and ACD. For example, HAVCR1 mediated 52.6 % of the relationship, whereas GDF15 mediated 22 % of the relationship. Other notable proteins included COL6A3, IGFBP4, LGALS4, CHI3L1, MMP12, ACE2, and IL-6. These proteins are involved in immune reactions, cytokine activity, and inflammatory cell migration. HAVCR1 (TIM-1), which features an immunoglobulin-like domain, activates the ERK and JNK pathways to regulate cell survival, apoptosis, and the release of inflammatory cytokines. Although few studies have investigated the role of HAVCR1 in dementia, a recent Mendelian randomization study provided pioneering genetic evidence that elevated HAVCR1 expression may causally contribute to ACD, AD, and VaD [32]. This finding positions HAVCR1 as a novel etiological player in dementia, potentially bridging its established roles in immune dysregulation and vascular inflammation with other neurodegenerative mechanisms. GDF15 serves as a crucial marker for predicting dementia onset years in advance [19]. Its upregulation in the central nervous system likely stems from vascular brain injuries, including stroke and cerebral microvascular diseases [33,34]. IL-6, which is elevated in inflammatory DJDs, may disrupt the blood–brain barrier (BBB), activate microglia, accelerate Aβ aggregation, and exacerbate tau hyperphosphorylation via astrocyte *trans*-signaling [[35], [36], [37], [38]]. MMP12, a matrix metalloproteinase, may disrupt the extracellular matrix (ECM) and contribute to BBB leakage [39]. In dementia, ACE2 deficiency exacerbates endothelial dysfunction and cerebral hypoperfusion, contributing to VaD [40]. CHI3L1, which is highly expressed in degenerative joints [41], contributes to dementia and neuroinflammation primarily by modulating microglial and astrocyte activity, driving pro-inflammatory cytokine release, and exacerbating neuroinflammation [42]. The identification of ECM-related proteins, such as COL6A3 and IGFBP4, suggests that extracellular matrix dysregulation in DJDs may also reflect or influence similar pathological remodeling in the brain vasculature and glial niches. Mediation analysis suggested that these factors were responsible for a significant proportion of the total effect of DJDs on dementia risk, indicating that systemic inflammation and immune signaling are not only correlated but also potential causal intermediates. These findings highlight the potential of these proteins as biomarkers for disease prediction and therapeutic targeting in the future.

Despite its strengths, this study has some limitations. First, although the UK Biobank provides a large and diverse dataset, it remains an observational study with inherent limitations. Causality cannot be definitively inferred, and residual confounding factors, such as unmeasured lifestyle factors or medication use, may have influenced these associations. Second, DJD diagnoses were based on clinical coding and self-reports rather than standardized clinical evaluations or imaging, which may have introduced diagnostic misclassification. Moreover, the present study lacked detailed information on DJD severity. Measures such as radiographic grading, or functional limitation indices were not available, which precluded us from examining whether the degree of DJD severity influenced the risk of dementia. Disease severity may reflect differences in chronic inflammation, physical inactivity, and systemic metabolic alterations, all of which may contribute to neurodegenerative processes. Future studies using more refined case definitions or radiographic confirmation could improve phenotypic precision and clarify how varying levels of DJD severity modulate the observed association between DJD and dementia risk. Third, although the proteomic data were of high quality and longitudinally linked, they were limited to the baseline measurements. Temporal changes in protein expression in response to disease progression or treatment have not been evaluated. Longitudinal proteomic profiling could elucidate dynamic mediating roles and identify time-dependent biomarkers. Finally, this study mainly focused on ACD, AD, and VaD as outcomes without subtyping them into Lewy body dementia. Future studies stratifying dementia types may reveal subtype-specific mechanisms and mediators of these relationships.

In conclusion, this study provides evidence of a link between DJDs and an increased risk of dementia, which is partly mediated by systemic protein signatures. Our findings may offer new insights into the development of early risk assessment, preventive interventions, and treatment strategies for dementia. We also highlighted the importance of incorporating cognitive monitoring into DJD management.

## Data availability

The data supporting this study’s findings are available on the UK Biobank at http://www.ukbiobank.ac.uk/. The code generated or used in this study is available upon reasonable requests.

## Funding Sources

S-MD was supported by the National Natural Science Foundation of China (Grant No. 82271837). ZK was supported by the Postdoctoral Fellowship Program of CPSF (Grant No. 2023M742331) and the Pujiang Rheumatism Young Physicians Training Program (Grant No. SPROG2302). CZ was supported by the National Natural Science Foundation of China (Grant No. 72573164), the Pinduoduo-China Agricultural University Research Fund (Grant No. PC2024B02011), and the 2115 Talent Development Program at China Agricultural University. QT was supported by the Basic Research Project of Shanghai Sixth People’s Hospital (Grant No. ynms202408).

## Author contributions

ZK, QT, and S-MD have full access to all the data in the study and take responsibility for the integrity and accuracy of the data analysis. QT and S-MD are joint corresponding authors. All authors have read the manuscript, provided critical feedback on the intellectual content, and approved the final version. Conceptualization: S-MD. Methodology: ZK, CZ, YZ, PL, and KL. Software: ZK. Data curation: ZK, ZC and PL. Investigation: ZK, JZ, CZ, YZ, PL, and KL. Validation: JZ and CZ. Formal analysis: ZK and JZ. Supervision: QT and S-MD. Funding acquisition: S-MD, ZK, QT and CZ. Visualization: ZK. Project administration: S-MD. Resources: QT. Writing – original draft: ZK. Writing – review & editing: JZ, YZ, PL, KL, CZ, EL, QT, and S-MD.

## Ethical approval

*The UK Biobank obtained ethical approval from the North West Multi-centre Research Ethics Committee (Reference number: 11/NW/0382,* *https://www.ukbiobank.ac.uk/learn-more-about-uk-biobank/about-us/ethics**). Informed consent was obtained from all the study participants via electronic signatures.*

## Declaration of competing interest

*The authors declare that they have no known competing financial interests or personal relationships that could have appeared to influence the work reported in this paper.*
