# Supplementary material for: Plasma proteomics mediate the association between degenerative joint diseases and dementia risk
Source: J Adv Res. 2025 Dec 28;88:795–806. doi: 10.1016/j.jare.2025.12.044 (PMC13669547; doi:10.1016/j.jare.2025.12.044)
Supplement: Supplementary file 1 [file mmc1.docx]

**Supplementary Materials**

**Plasma Proteomics Mediate the Association Between Degenerative Joint Diseases and Dementia Risk**

Zijian Kang, MD^1#^, Jianzheng Zhang, MD, PhD^2#^, Chen Zhu, PhD^3#^, Edward CH Lau^4^, Ying Zhu, MD, PhD^5^, Ping Li, PhD^6^, Kai Li, PhD^6^, Qiang Tong, MD, PhD^1,4*^ and Sheng-Ming Dai, MD, PhD^1*^

1. Department of Rheumatology and Immunology, Shanghai Sixth People’s Hospital Affiliated to Shanghai Jiao Tong University School of Medicine, Shanghai, China
2. Senior Department of Orthopedics, The Forth Medical Center of Chinese PLA General Hospital, Beijing, China
3. College of Economics and Management, China Agricultural University, Beijing, China
4. Division of Rheumatology and Clinical Immunology, Department of Medicine, Queen Mary Hospital, University of Hong Kong, Hong Kong, China
5. Senior Department of Respiratory and Critical Care Medicine, The Eighth Medical Center of Chinese PLA General Hospital, Beijing, China
6. College of Biological Science and Medical Engineering, Donghua University, Shanghai, China

#These authors contributed equally to this study.

*Corresponding author:

Prof. Sheng-Ming Dai, Department of Rheumatology and Immunology, Shanghai Sixth People's Hospital Affiliated to Shanghai Jiao Tong University School of Medicine, Shanghai 200233, China ([shengmingdai@163.com](mailto:shengmingdai@163.com))

Dr. Qiang Tong, Department of Rheumatology and Immunology, Shanghai Sixth People's Hospital Affiliated to Shanghai Jiao Tong University School of Medicine, Shanghai 200233, China (jasontong1985@outlook.com)

**Appendices index**

[**Supplement Figure 1. Flowchart of participant selection.**	4](#_Toc214123042)

[**Supplement Figure 2. Pie charts illustrating the proportion of data resources for DJD and ACD.**	5](#_Toc214123043)

[**Supplement Figure 3. Sensitivity analyses of the association between DJD and ACD risk**.	6](#_Toc214123044)

[**Supplement Figure 4. Sensitivity analyses of the association between DJD subtypes and ACD risk**.	7](#_Toc214123045)

[**Supplement Figure 5. Subgroup analysis of the association between DJD and ACD risk**.	8](#_Toc214123046)

[**Supplement Figure 6. Subgroup analysis of the association between OA and ACD risk.**	9](#_Toc214123047)

[**Supplement Figure 7. Subgroup analysis of the association between IVDD and ACD risk**.	10](#_Toc214123048)

[**Supplement Figure 8. Subgroup analysis of the association between DSD and ACD risk**.	11](#_Toc214123049)

[**Supplement Figure 9. Joint association of DJD status with the risk of ACD, stratified by activity level and surgical procedure.**	12](#_Toc214123050)

[**Supplement Figure 10. Proteins associated with DJD identified through multiple linear regression models.**	13](#_Toc214123051)

[**Supplement Figure 11. Functional enrichment analysis of DJD-associated proteins.**	14](#_Toc214123052)

[**Supplement Table 1. Codes used for DJD and dementia diagnosis and classification in the UK Biobank**	15](#_Toc214123053)

[**Supplement Table 2. Schoenfeld residuals test in Cox proportional hazards model**	16](#_Toc214123054)

[**Supplement Table 3. Multivariable Cox regression analysis of the association between DJD and incident ACD, treating incident DJD cases as a time-varying exposure**	17](#_Toc214123055)

[**Supplement Table 4. Multivariable Cox regression analysis of the association between DJD and incident AD, treating incident DJD cases as a time-varying exposure**	18](#_Toc214123056)

[**Supplement Table 5. Multivariable Cox regression analysis of the association between DJD and incident VaD, treating incident DJD cases as a time-varying exposure**	19](#_Toc214123057)

[**Supplement Table 6. Multivariable Cox regression analysis for incident ACD, employing a censoring approach for incident DJD cases occurring during follow-up**	20](#_Toc214123058)

[**Supplement Table 7. Multivariable Cox regression analysis for incident ACD, employing a excluding approach for incident DJD cases occurring during follow-up**	21](#_Toc214123059)

[**Supplement Table 8. Multivariable Cox regression analysis for incident VaD, employing a censoring approach for incident DJD cases occurring during follow-up**	22](#_Toc214123060)

[**Supplement Table 9. Multivariable Cox regression analysis for incident VaD, employing a excluding approach for incident DJD cases occurring during follow-up**	23](#_Toc214123061)

[**Supplement Table 10. Sensitivity analyses for association between DJDs and ACD with further adjustment for surgical procedure**	24](#_Toc214123062)

[**Supplement Table 11. Sensitivity analyses of the association between DJD and incident ACD by excluding ACD with follow-up duration <2 years**	25](#_Toc214123063)

[**Supplement Table 12. Sensitivity analyses of the association between DJD and incident ACD by excluding ACD diagnosed before age 65.**	26](#_Toc214123064)

[**Supplement Table 13. Fine-Grey analysis of the risk of DJD and incident ACD**	27](#_Toc214123065)

[**Supplement Table 14. Multivariable Cox regression for ACD risk stratified by the presence or absence of pain among participants with DJD**	28](#_Toc214123066)

[**Supplement Table 15. Standardized mean differences before and after matching for the DJD Cohort**	29](#_Toc214123067)

[**Supplement Table 16. PSM analysis of the risk of DJD and incident ACD**	30](#_Toc214123068)

[**Supplement Table 17. Identification of top DJD-associated proteins using generalized linear regression**	31](#_Toc214123069)

[**Supplement Table 18. GO, KEGG and Reactome enrichment analysis of DJD-associated proteins**	34](#_Toc214123070)

[**Supplement Table 19. Identification of top OA-associated proteins using generalized linear regression**	37](#_Toc214123071)

[**Supplement Table 20. Identification of top IVDD-associated proteins using generalized linear regression**	40](#_Toc214123072)

[**Supplement Table 21. Identification of top DSD-associated proteins using generalized linear regression**	43](#_Toc214123073)

[**Supplement Table 22. GO, KEGG enrichment analysis of DJD subtype-associated proteins**	46](#_Toc214123074)

[**Supplement Table 23. Identification of ACD-associated proteins using multivariable Cox regression**	52](#_Toc214123075)

[**Supplement Table 24. GO, KEGG and Reactome enrichment analysis of ACD-associated proteins**	55](#_Toc214123076)

**Supplement Figure 1. Flowchart of participant selection.**

In the UK Biobank cohort, individuals who reported three types of DJD at baseline or those without DJD were included in the cohort study. In the mediation analysis, individuals with proteomic data were further performed mediation analysis.

**Abbreviations:** DJD: Degenerative joint disease; OA: Osteoarthritis; IVDD: Intervertebral disc degeneration; DSD: Degenerative spinal diseases; ACD: All-cause dementia; AD: Alzheimer's disease; VaD: Vascular dementia.

**Supplement Figure 2. Pie charts illustrating the proportion of data resources for DJD and ACD.**

**Supplement Figure 3. Sensitivity analyses of the association between DJD and ACD risk**.

(A-B). Cumulative incidence curves using the Fine-Gray competing risk model. Cumulative incidence curves show the cumulative incidence of ACD (A) and death (B), with death considered a competing risk. The red and blue lines represent the DJD and control groups, respectively. The numbers at risk and events are provided below each plot to indicate the sample size and event occurrence at different follow-up intervals.

**Supplement Figure 4. Sensitivity analyses of the association between DJD subtypes and ACD risk**.

(A-C). Cumulative incidence curves using the Fine-Gray competing risk model. Cumulative incidence curves show the cumulative incidence of ACD and death, with death considered a competing risk in OA (A), IVDD (B), and DSD (C). The red and blue lines represent the exposure and control groups, respectively. The numbers at risk and events are provided below each plot to indicate the sample size and event occurrence at different follow-up intervals.

**Supplement Figure 5. Subgroup analysis of the association between DJD and ACD risk**.

The forest plot showing the HRs and 95% CIs for the association between DJD and ACD risk across various subgroups. The subgroups were based on demographic factors (age, sex, ethnicity), socioeconomic status (Townsend index), lifestyle factors (smoking, drinking, activity level), comorbidities (hypertension, diabetes, CVD), and surgical procedures. The P-values for interaction are provided to assess the heterogeneity of the association across subgroups. The analysis was based on multivariate Cox regression models.

**Abbreviations:** MET: Metabolic equivalent task; HR: hazard ratios; CI: confidence intervals.

**Supplement Figure 6. Subgroup analysis of the association between OA and ACD risk.**

The forest plot showing the HRs and 95% CIs for the association between OA and ACD risk across various subgroups. The subgroups were based on demographic factors (age, sex, ethnicity), socioeconomic status (Townsend index), lifestyle factors (smoking, drinking, activity level), comorbidities (hypertension, diabetes, CVD), and surgical procedures. The P-values for interaction are provided to assess the heterogeneity of the association across subgroups. The analysis was based on multivariate Cox regression models.

**Supplement Figure 7. Subgroup analysis of the association between IVDD and ACD risk**.

The forest plot presents the HRs and 95% CIs for the association between IVDD and ACD risk across various subgroups. The subgroups were based on demographic factors (age, sex, ethnicity), socioeconomic status (Townsend index), lifestyle factors (smoking, drinking, activity level), comorbidities (hypertension, diabetes, CVD), and surgical procedures. The P-values for interaction are provided to assess the heterogeneity of the association across subgroups. The analysis was based on multivariate Cox regression models.

**Supplement Figure 8. Subgroup analysis of the association between DSD and ACD risk**.

The forest plot presents the HRs and 95% CIs for the association between IVDD and ACD risk across various subgroups. The subgroups were based on demographic factors (age, sex, ethnicity), socioeconomic status (Townsend index), lifestyle factors (smoking, drinking, activity level), comorbidities (hypertension, diabetes, CVD), and surgical procedures. The P-values for interaction are provided to assess the heterogeneity of the association across subgroups. The analysis was based on multivariate Cox regression models.

**Supplement Figure 9. Joint association of DJD status with the risk of ACD, stratified by activity level and** **surgical procedure.**

**(A-D).** Forest plots of HRs and 95% CIs for ACD according to DJD (A), OA (B), IVDD (C), and DSD (D), stratified by activity level.

**(E-F).** Forest plots of HRs and 95% CIs for ACD according to DJD (E) and OA (F), stratified by surgical procedure.

**Supplement Figure 10. Proteins associated with DJD identified through multiple linear regression models.**

A-B. Dot plots showing proteins positively (A) and negatively (B) associated with DJD. Each dot represents a protein, with the color intensity indicating the beta value and the size of the dot reflecting the FDR-adjusted p-value. Proteins were mapped across different DJD subtypes (OA, IVDD, and DSD).

**Abbreviations:** FDR: False discovery rate.

**Supplement Figure 11. Functional enrichment analysis of DJD-associated proteins.**

A-B. GO (A) and KEGG (B) enrichment analysis illustrating the signaling pathways enriched in DJD-related proteins. The size of the dots represents the gene count ratio (GeneRatio), and the color intensity indicates the adjusted p-value. Analyses were performed using multiple linear regression models.

**Abbreviations:** GO: Gene Ontology; KEGG: Kyoto Encyclopedia of Genes and Genomes.

**Supplement Table 1. Codes used for DJD and dementia diagnosis and classification in the UK Biobank**

| **Disease** | **ICD-10 codes** |
| --- | --- |
| **OA** | M15-M19 |
| **IVDD** | M51 |
| **DSD** | M41, M43, M47, M48, M53 |
| **ACD** | F00-F03, G30 |
| **AD** | G30, F00 |
| **VaD** | F01 |

**Abbreviations:** ICD: International Classification of Diseases**;** DJD: Degenerative joint disease; OA: Osteoarthritis; IVDD: Intervertebral disc degeneration; DSD: Degenerative spinal diseases; ACD: All-cause dementia; AD: Alzheimer's disease; VaD: Vascular dementia.

**Supplement Table 2.** **Schoenfeld residuals test in Cox proportional hazards model**

| **Variable** | **Chi-square** | **df** | **P-value** |
| --- | --- | --- | --- |
| DJD status | 0.465 | 1 | 0.495 |
| Age | 0.618 | 1 | 0.432 |
| Sex | 1.437 | 1 | 0.231 |
| Ethnicity | 0.978 | 1 | 0.323 |
| Educational qualification | 5.669 | 4 | 0.225 |
| Townsend | 2.192 | 1 | 0.139 |
| Smoking | 0.167 | 2 | 0.920 |
| Drinking | 1.162 | 1 | 0.281 |
| MET level | 3.534 | 3 | 0.316 |
| Hypertension | 2.380 | 1 | 0.123 |
| Diabetes | 1.902 | 1 | 0.168 |
| CVD | 0.451 | 1 | 0.502 |
| GLOBAL | 23.835 | 18 | 0.161 |

**Abbreviations**: A levels/AS levels: Advanced levels/Advanced Subsidiary levels; O levels/GCSEs: Ordinary levels/General Certificate of Secondary Education; CSEs or equivalent: Certificate of Secondary Education or equivalent; NVQ or HND or HNC: National Vocational Qualification or Higher National Diploma or Higher National Certificate.

**Supplement Table 3. Multivariable Cox regression analysis of the association between DJD and incident ACD, treating incident DJD cases as a time-varying exposure**

| **Group** | **Incidence (cases/person-years)** | **HR (95% CI)** | | | |
| --- | --- | --- | --- | --- | --- |
|  |  | **Model 0** | **Model 1** | **Model 2** | **Model 3** |
| **Control** | 5274/5149168 | Ref | Ref | Ref | Ref |
| **(N=406760)** |  |  |  |  |  |
| **ALL DJD** | 4610/1869325 | 2.087 (2.005–2.171) | 1.368 (1.314–1.424) | 1.286 (1.211–1.367) | 1.271 (1.173–1.376) |
| **(N=94045)** |  |  |  |  |  |
| **OA** | 3433/1327391 | 2.068 (1.982–2.158) | 1.268 (1.214–1.324) | 1.180 (1.105–1.260) | 1.177 (1.079–1.285) |
| **(N=64482)** |  |  |  |  |  |
| **IVDD** | 702/311320 | 1.749(1.619–1.891) | 1.508 (1.394–1.631) | 1.451 (1.292–1.629) | 1.488 (1.279–1.730) |
| **(N=** **17401)** |  |  |  |  |  |
| **DSD** | 1543/525513 | 2.329 (2.203–2.462) | 1.614 (1.526–1.708) | 1.515 (1.388–1.654) | 1.569 (1.400–1.760) |
| **(N=** **27841)** |  |  |  |  |  |

**Abbreviations:** HR: Hazard ratio; CI: Confidence interval.

**Supplement Table 4.** **Multivariable Cox regression analysis of the association between DJD and incident AD, treating incident DJD cases as a time-varying exposure**

| **Group** | **Incidence (cases/person-years)** | **HR (95% CI)** | | | |
| --- | --- | --- | --- | --- | --- |
|  |  | **Model 0** | **Model 1** | **Model 2** | **Model 3** |
| **Control** | 2395/5156395 | Ref | Ref | Ref | Ref |
| **(N=406760)** |  |  |  |  |  |
| **ALL DJD** | 2009/1878570 | 1.956 (1.843–2.075) | 1.224 (1.152–1.300) | 1.109 (1.011–1.216) | 1.107 (0.982–1.248) |
| **(N=94045)** |  |  |  |  |  |
| **OA** | 1558/1334164 | 2.060 (1.933–2.195) | 1.207 (1.131–1.287) | 1.085 (0.982–1.199) | 1.107 (0.973–1.260) |
| **(N=64482)** |  |  |  |  |  |
| **IVDD** | 291/312904 | 1.591 (1.411–1.795) | 1.358 (1.203–1.532) | 1.211 (1.004–1.462) | 1.185 (0.926–1.516) |
| **(N=** **17401)** |  |  |  |  |  |
| **DSD** | 626/528836 | 2.047 (1.877–2.232) | 1.357 (1.244–1.481) | 1.235 (1.073–1.421) | 1.253 (1.045–1.502) |
| **(N=** **27841)** |  |  |  |  |  |

**Supplement Table 5. Multivariable Cox regression analysis of the association between DJD and incident VaD, treating incident DJD cases as a time-varying exposure**

| **Group** | **Incidence (cases/person-years)** | **HR (95% CI)** | | | |
| --- | --- | --- | --- | --- | --- |
|  |  | **Model 0** | **Model 1** | **Model 2** | **Model 3** |
| **Control** | 1088/5159568 | Ref | Ref | Ref | Ref |
| **(N=406760)** |  |  |  |  |  |
| **ALL DJD** | 1136/1881510 | 2.490 (2.291–2.707) | 1.616 (1.485–1.759) | 1.496 (1.315–1.702) | 1.587 (1.323–1.903) |
| **(N=94045)** |  |  |  |  |  |
| **OA** | 851/1336546 | 2.434 (2.229–2.658) | 1.475 (1.349–1.613) | 1.346 (1.171–1.547) | 1.358 (1.116–1.653) |
| **(N=64482)** |  |  |  |  |  |
| **IVDD** | 169/313270 | 1.927 (1.643–2.260) | 1.644 (1.401–1.930) | 1.814 (1.445–2.276) | 2.156 (1.603–2.899) |
| **(N=17401)** |  |  |  |  |  |
| **DSD** | 398/529589 | 2.791 (2.496–3.122) | 1.924 (1.719–2.154) | 1.660 (1.382–1.993) | 1.838 (1.433–2.356) |
| **(N=27841)** |  |  |  |  |  |

**Supplement Table 6. Multivariable Cox regression analysis for incident ACD, employing a censoring approach for incident DJD cases occurring during follow-up**

| **Group** | **Incidence (cases/person-years)** | **HR (95% CI)** | | | |
| --- | --- | --- | --- | --- | --- |
|  |  | **Model 0** | **Model 1** | **Model 2** | **Model 3** |
| **Control** | 5274/5709759 | Ref | Ref | Ref | Ref |
| **(N=406760)** |  |  |  |  |  |
| **ALL DJD** | 2973/1300158 | 2.493 (2.383–2.608) | 1.670 (1.596–1.748) | 1.499 (1.397–1.608) | 1.485 (1.352–1.630) |
| **(N=94045)** |  |  |  |  |  |
| **OA** | 2226/889182 | 2.530 (2.409–2.657) | 1.552 (1.476–1.631) | 1.386 (1.282–1.499) | 1.376 (1.240–1.528) |
| **(N=64482)** |  |  |  |  |  |
| **IVDD** | 460/241553 | 1.640 (1.492–1.802) | 1.393 (1.267–1.532) | 1.374 (1.196–1.578) | 1.461 (1.223–1.745) |
| **(N=** **17401)** |  |  |  |  |  |
| **DSD** | 969/383063 | 2.302 (2.152–2.463) | 1.586 (1.482–1.698) | 1.405 (1.257–1.570) | 1.455 (1.259–1.682) |
| **(N=** **27841)** |  |  |  |  |  |

**Supplement Table 7. Multivariable Cox regression analysis for incident ACD, employing a excluding approach for incident DJD cases occurring during follow-up**

| **Group** | **Incidence (cases/person-years)** | **HR (95% CI)** | | | |
| --- | --- | --- | --- | --- | --- |
|  |  | **Model 0** | **Model 1** | **Model 2** | **Model 3** |
| **Control** | 5274/5700765 | Ref | Ref | Ref | Ref |
| **(N=405123)** |  |  |  |  |  |
| **ALL DJD** | 2973/1300158 | 2.491 (2.382–2.606) | 1.667 (1.592–1.744) | 1.496 (1.395–1.605) | 1.481 (1.350–1.626) |
| **(N=94045)** |  |  |  |  |  |
| **OA** | 2226/889182 | 2.528 (2.407–2.655) | 1.549 (1.474–1.628) | 1.384 (1.28–1.497) | 1.374 (1.238–1.525) |
| **(N=64482)** |  |  |  |  |  |
| **IVDD** | 460/241553 | 1.640 (1.492–1.802) | 1.392 (1.266–1.531) | 1.374 (1.196–1.578) | 1.461 (1.223–1.744) |
| **(N=** **17401)** |  |  |  |  |  |
| **DSD** | 969/383063 | 2.301 (2.151–2.462) | 1.585 (1.480–1.697) | 1.404 (1.256–1.568) | 1.454 (1.257–1.681) |
| **(N=** **27841)** |  |  |  |  |  |

**Supplement Table 8. Multivariable Cox regression analysis for incident VaD, employing a censoring approach for incident DJD cases occurring during follow-up**

| **Group** | **Incidence (cases/person-years)** | **HR (95% CI)** | | | |
| --- | --- | --- | --- | --- | --- |
|  |  | **Model 0** | **Model 1** | **Model 2** | **Model 3** |
| **Control** | 1088/5159568 | Ref | Ref | Ref | Ref |
| **(N=****406760)** |  |  |  |  |  |
| **ALL DJD** | 1136/1881510 | 2.870 (2.611–3.155) | 1.905 (1.731–2.097) | 1.695 (1.459–1.969) | 1.798 (1.458–2.218) |
| **(N=94045)** |  |  |  |  |  |
| **OA** | 851/1336546 | 2.885 (2.606–3.194) | 1.750 (1.579–1.941) | 1.500(1.270–1.771) | 1.549 (1.227–1.955) |
| **(N=64482)** |  |  |  |  |  |
| **IVDD** | 169/313270 | 1.729 (1.420–2.106) | 1.451 (1.190–1.769) | 1.698 (1.294–2.227) | 2.162 (1.531–3.054) |
| **(N=** **17401)** |  |  |  |  |  |
| **DSD** | 398/529590 | 2.661 (2.320–3.052) | 1.829 (1.593–2.099) | 1.450 (1.144–1.837) | 1.592 (1.153–2.198) |
| **(N=** **27841)** |  |  |  |  |  |

**Supplement Table 9. Multivariable Cox regression analysis for incident VaD, employing a excluding approach for incident DJD cases occurring during follow-up**

| **Group** | **Incidence (cases/person-years)** | **HR (95% CI)** | | | |
| --- | --- | --- | --- | --- | --- |
|  |  | **Model 0** | **Model 1** | **Model 2** | **Model 3** |
| **Control** | 1088/5159568 | Ref | Ref | Ref | Ref |
| **(N=405123)** |  |  |  |  |  |
| **ALL DJD** | 1136/1881510 | 2.869 (2.610–3.154) | 1.904 (1.730–2.095) | 1.694 (1.458–1.968) | 1.797 (1.456–2.217) |
| **(N=94045)** |  |  |  |  |  |
| **OA** | 851/1336546 | 2.885 (2.606–3.194) | 1.749 (1.578–1.939) | 1.499 (1.269–1.771) | 1.548 (1.226–1.954) |
| **(N=64482)** |  |  |  |  |  |
| **IVDD** | 169/313270 | 1.729 (1.420–2.106) | 1.451 (1.190–1.769) | 1.697 (1.294–2.227) | 2.162 (1.530–3.053) |
| **(N=** **17401)** |  |  |  |  |  |
| **DSD** | 398/529590 | 2.661 (2.320–3.052) | 1.828 (1.592–2.099) | 1.449 (1.144–1.836) | 1.591 (1.152–2.197) |
| **(N=** **27841)** |  |  |  |  |  |

**Supplement Table 10. Sensitivity analyses for association between DJDs and ACD with further adjustment for surgical procedure**

|  | **HR (95% CI)** | P value |
| --- | --- | --- |
| **Control** | 1 | ref |
| **(N=406760)** |  |  |
| **ALL DJD** | 1.270 (1.170–1.379) | <0.001 |
| **(N=94045)** |  |  |
| **OA** | 1.215 (1.204–1.278) | <0.001 |
| **(N=64482)** |  |  |
| **IVDD** | 1.504 (1.288–1.756) | <0.001 |
| **(N=** **17401)** |  |  |
| **DSD** | 1.563 (1.392–1.755) | <0.001 |
| **(N=** **27841)** |  |  |

**Supplement Table 11. Sensitivity analyses of the association between DJD and incident ACD by excluding ACD with follow-up duration <2 years**

| **Group** | **Incidence (cases/person-years)** | **HR (95% CI)** | | | |
| --- | --- | --- | --- | --- | --- |
|  |  | **Model 0** | **Model 1** | **Model 2** | **Model 3** |
| **Control** | 5167/5149031 | Ref | Ref | Ref | Ref |
| **(N=406641)** |  |  |  |  |  |
| **ALL DJD** | 4556/1869271 | 2.088(2.006–2.173) | 1.366(1.312–1.423) | 1.285(1.209–1.365) | 1.267(1.169–1.373) |
| **(N=94003)** |  |  |  |  |  |
| **OA** | 3394/1327351 | 2.068(1.982–2.159) | 1.266(1.212–1.322) | 1.178(1.103–1.259) | 1.174(1.075–1.281) |
| **(N=64450)** |  |  |  |  |  |
| **IVDD** | 694/311312 | 1.752(1.62–1.895) | 1.51(1.396–1.633) | 1.450(1.29–1.63) | 1.470(1.262–1.712) |
| **(N=** **17396)** |  |  |  |  |  |
| **DSD** | 1523/525493 | 2.328(2.201–2.462) | 1.612(1.523–1.706) | 1.506(1.378–1.645) | 1.557(1.388–1.747) |
| **(N=** **27825)** |  |  |  |  |  |

**Supplement Table 12. Sensitivity analyses of the association between DJD and incident ACD by excluding ACD diagnosed before age 65.**

| **Group** | **Incidence (cases/person-years)** | **HR (95% CI)** | | | |
| --- | --- | --- | --- | --- | --- |
|  |  | **Model 0** | **Model 1** | **Model 2** | **Model 3** |
| **Control** | 4831/5145661 | Ref | Ref | Ref | Ref |
| **(N=406215)** |  |  |  |  |  |
| **ALL DJD** | 4325/1867769 | 2.111 (2.026–2.200) | 1.316 (1.262–1.372) | 1.227 (1.152–1.307) | 1.199 (1.103–1.303) |
| **(N=93862)** |  |  |  |  |  |
| **OA** | 3249/1326416 | 2.111 (2.021–2.206) | 1.227 (1.174–1.284) | 1.137 (1.062–1.217) | 1.119 (1.022–1.225) |
| **(N=64361)** |  |  |  |  |  |
| **IVDD** | 635/310945 | 1.705 (1.571–1.850) | 1.442 (1.328–1.565) | 1.382 (1.222–1.562) | 1.400 (1.192–1.643) |
| **(N=** **17360)** |  |  |  |  |  |
| **DSD** | 1451/525005 | 2.364 (2.232–2.504) | 1.569 (1.480–1.663) | 1.470 (1.342–1.610) | 1.521(1.350–1.712) |
| **(N=** **27779)** |  |  |  |  |  |

**Supplement Table 13. Fine-Grey analysis of the risk of DJD and incident ACD**

| **Exposure** | **Hazard Ratio** | **2.50%** | **97.50%** | **P value** |
| --- | --- | --- | --- | --- |
| **Control** | 1 | 1 | 1 | Reference |
| **DJD** | 1.632 | 1.427 | 1.865 | 7.00E-13 |
| **OA** | 1.461 | 1.257 | 1.698 | 8.00E-07 |
| **IVDD** | 1.555 | 1.211 | 1.998 | 0.00055 |
| **DSD** | 1.548 | 1.263 | 1.898 | 2.60E-05 |

| **Group** | **Incidence (cases/person-years)** | **HR (95% CI)** | | | |
| --- | --- | --- | --- | --- | --- |
|  |  | **Model 0** | **Model 1** | **Model 2** | **Model 3** |
| **Control** | 5274/5709758 | Ref | Ref | Ref | Ref |
| **(N=406760)** |  |  |  |  |  |
| **DSD-no-pain** | 964/478606 | 2.191 (2.046– 2.347) | 1.450 (1.353– 1.555) | 1.307 (1.174– 1.455) | 1.315 (1.142– 1.513) |
| **(N=34489)** |  |  |  |  |  |
| **DJD-pain** | 2009/821552 | 2.669 (2.536– 2.810) | 1.800 (1.708– 1.896) | 1.620 (1.493– 1.758) | 1.597 (1.432– 1.782) |
| **(N=59556)** |  |  |  |  |  |
| **OA-no-pian** | 679/305885 | 2.239 (2.068– 2.424) | 1.357 (1.252– 1.471) | 1.215 (1.070– 1.379) | 1.201 (1.016– 1.420) |
| **(N=22094)** |  |  |  |  |  |
| **OA-pain** | 1547/583297 | 2.683 (2.536– 2.838) | 1.655 (1.563– 1.752) | 1.483 (1.354– 1.624) | 1.480 (1.310– 1.672) |
| **(N=42388)** |  |  |  |  |  |
| **IVDD-no-pian** | 124/792231 | 1.344 (1.125– 1.606) | 1.092 (0.913– 1.306) | 1.120 (0.871– 1.44) | 1.208 (0.883– 1.653) |
| **(N=** **5678)** |  |  |  |  |  |
| **IVDD-pain** | 336/1623228 | 1.785 (1.600– 1.992) | 1.549 (1.388– 1.729) | 1.515 (1.287– 1.783) | 1.609 (1.303– 1.988) |
| **(N=** **27728)** |  |  |  |  |  |
| **DSD-no-pain** | 331/150918 | 1.990 (1.782– 2.223) | 1.380 (1.235– 1.543) | 1.173 (0.977– 1.409) | 1.284 (1.017– 1.621) |
| **(N= 10931)** |  |  |  |  |  |
| **DSD-pain** | 638/232145 | 2.506 (2.31– 2.718) | 1.719 (1.584– 1.866) | 1.565 (1.368– 1.789) | 1.573 (1.317– 1.879) |
| **(N= 16910)** |  |  |  |  |  |

**Supplement Table 14. Multivariable Cox regression for ACD risk stratified by the presence or absence of pain among participants with DJD**

| **Variables** | **PSM DJD cohort** | | **PSM OA cohort** | | **PSM IVD cohort** | | **PSM DSD cohort** | |
| --- | --- | --- | --- | --- | --- | --- | --- | --- |
|  | **N = 94,011** | | **N = 64,475** | | **N = 17,399** | | **N = 27,835** | |
|  | **Before** | **After** | **Before** | **After** | **Before** | **After** | **Before** | **After** |
| **Age** | 0.552 | -0.022 | 0.762 | -0.025 | 0.195 | -0.009 | 0.503 | -0.018 |
| **Sex (%)** |  |  |  |  |  |  |  |  |
| Female | 0.115 | 0.012 | 0.155 | 0.012 | -0.030 | 0.017 | 0.154 | 0.003 |
| Male | -0.115 | -0.012 | -0.155 | -0.012 | 0.030 | -0.017 | -0.154 | -0.003 |
| **Ethnicity (%)** |  |  |  |  |  |  |  |  |
| White | 0.094 | -0.045 | 0.104 | -0.05 | 0.079 | -0.06 | 0.094 | -0.054 |
| Non-white | -0.102 | 0.041 | -0.115 | 0.041 | -0.078 | 0.056 | -0.096 | 0.049 |
| **Educational Qualification (%)** | |  |  |  |  |  |  |  |
| College or University | -0.122 | 0.006 | -0.147 | 0.001 | -0.051 | 0.002 | -0.157 | 0.015 |
| A levels/AS levels | -0.034 | 0.012 | -0.043 | 0.011 | -0.012 | 0.018 | -0.036 | 0.020 |
| O levels/GCSEs | -0.080 | -0.001 | -0.094 | -0.005 | -0.060 | 0.007 | -0.081 | -0.001 |
| CSEs or equivalent | -0.022 | 0.006 | -0.035 | 0.006 | -0.004 | -0.005 | -0.019 | 0.011 |
| NVQ or HND or HNC | -0.046 | -0.005 | -0.056 | -0.005 | 0.010 | -0.005 | -0.087 | -0.008 |
| **Townsend (%)** |  |  |  |  |  |  |  |  |
| 1 (least deprived) | -0.037 | -0.014 | -0.048 | -0.011 | -0.006 | -0.005 | -0.057 | -0.009 |
| 2 | -0.006 | -0.010 | -0.012 | -0.011 | 0.007 | -0.011 | -0.016 | -0.007 |
| 3 | 0.003 | -0.001 | 0.004 | 0.003 | 0.001 | -0.002 | 0.007 | 0.001 |
| 4 (most deprived) | 0.039 | 0.023 | 0.054 | 0.018 | -0.001 | 0.016 | 0.061 | 0.012 |
| **Smoking (%)** |  |  |  |  |  |  |  |  |
| Never | -0.109 | 0.000 | -0.116 | 0.003 | -0.118 | 0.008 | -0.131 | 0.008 |
| Previous | 0.109 | -0.011 | 0.130 | -0.016 | 0.081 | -0.013 | 0.100 | -0.015 |
| Current | 0.002 | 0.012 | -0.024 | 0.017 | 0.060 | 0.001 | 0.048 | 0.006 |
| **Drinking (%)** |  |  |  |  |  |  |  |  |
| Never | 0.038 | 0.033 | 0.047 | 0.035 | 0.011 | 0.040 | 0.050 | 0.029 |
| Occasional | 0.075 | 0.033 | 0.080 | 0.039 | 0.076 | 0.025 | 0.106 | 0.043 |
| Frequent | -0.081 | -0.049 | -0.092 | -0.055 | -0.062 | -0.048 | -0.112 | -0.054 |
| **Activity level (%)** |  |  |  |  |  |  |  |  |
| Never | 0.070 | 0.027 | 0.072 | 0.033 | 0.097 | 0.010 | 0.099 | 0.016 |
| Low | -0.017 | 0.013 | -0.027 | 0.016 | 0.024 | 0.008 | -0.002 | 0.011 |
| Medium | -0.112 | -0.010 | -0.129 | -0.010 | -0.084 | -0.005 | -0.133 | -0.003 |
| High | -0.009 | -0.014 | -0.011 | -0.015 | -0.009 | -0.009 | -0.027 | -0.014 |
| **Hypertension (%)** |  |  |  |  |  |  |  |  |
| No | -0.178 | 0.003 | -0.235 | 0.003 | -0.062 | -0.005 | -0.158 | -0.005 |
| Yes | 0.197 | -0.007 | 0.253 | -0.009 | 0.095 | 0.001 | 0.176 | -0.004 |
| **Diabetes (%)** |  |  |  |  |  |  |  |  |
| No | -0.017 | -0.047 | -0.030 | -0.048 | 0.005 | -0.057 | -0.007 | -0.049 |
| Yes | 0.058 | 0.038 | 0.072 | 0.040 | 0.035 | 0.044 | 0.054 | 0.037 |
| **CVD (%)** |  |  |  |  |  |  |  |  |
| No | -0.223 | -0.018 | -0.269 | -0.019 | -0.144 | -0.015 | -0.231 | -0.017 |
| Yes | 0.124 | 0.026 | 0.140 | 0.034 | 0.097 | 0.017 | 0.158 | 0.022 |

**Supplement Table 15. Standardized mean differences before and after matching for the DJD Cohort**

**Supplement Table 16. PSM analysis of the risk of DJD and incident ACD**

| **Group** | **Model 1** | | **Model 2** | | **Model 3** | |
| --- | --- | --- | --- | --- | --- | --- |
|  | **HR (95% CI)** | **P value** | **HR (95% CI)** | **P value** | **HR (95% CI)** | **P value** |
| **Control** | 1 | ref | 1 | ref | 1 | ref |
| **DJD** | 1.567 (1.480–1.660) | 4.81E-53 | 1.511 (1.427–1.600) | 8.10E-46 | 1.494 (1.411–1.581) | 1.26E-43 |
| **OA** | 1.560 (1.460–1.667) | 1.66E-39 | 1.478 (1.384–1.577) | 1.17E-31 | 1.464 (1.372–1.563) | 2.25E-30 |
| **IVDD** | 1.654 (1.426–1.918) | 3.09E-11 | 1.577 (1.362–1.326) | 1.09E-09 | 1.457 (1.262–1.681) | 2.74E-07 |
| **DSD** | 1.736 (1.565–1.926) | 7.53E-09 | 1.622 (1.465–1.796) | 1.50E-04 | 1.748 (1.575–1.940) | 9.40E-26 |

**Model 1:** Matched for age, sex, and ethnicity.

**Model 2:** Further matched for education qualification, Townsend deprivation index, smoking behavior, alcohol consumption, and physical activity.

**Model 3:** Matched for all covariates from Model 2, plus hypertension, diabetes, and CVD.

**Abbreviations:** PSM: Propensity score matching.

**Supplement Table 17. Identification of top DJD-associated proteins using generalized linear regression**

| **Proteins** | **Beta** | **CI_lower** | **CI_upper** | **pvalue** | **FDR** |
| --- | --- | --- | --- | --- | --- |
| GAST | 0.245 | 0.217 | 0.272 | 1.90E-68 | 5.55E-66 |
| LEP | 0.228 | 0.204 | 0.252 | 1.72E-78 | 8.38E-76 |
| COL9A1 | 0.165 | 0.153 | 0.176 | 5.04E-183 | 1.47E-179 |
| FABP4 | 0.151 | 0.136 | 0.166 | 3.48E-84 | 2.54E-81 |
| CHGA | 0.146 | 0.128 | 0.164 | 1.42E-55 | 1.66E-53 |
| SSC4D | 0.137 | 0.107 | 0.167 | 5.40E-19 | 5.30E-18 |
| KRT18 | 0.130 | 0.109 | 0.15 | 4.10E-35 | 1.14E-33 |
| FGF21 | 0.126 | 0.095 | 0.156 | 8.01E-16 | 6.50E-15 |
| IL1RN | 0.125 | 0.11 | 0.14 | 5.85E-61 | 1.14E-58 |
| FABP1 | 0.122 | 0.099 | 0.146 | 5.12E-24 | 7.27E-23 |
| OXT | 0.122 | 0.086 | 0.159 | 3.03E-11 | 1.72E-10 |
| IL6 | 0.118 | 0.099 | 0.136 | 1.98E-34 | 5.20E-33 |
| CDCP1 | 0.116 | 0.103 | 0.128 | 1.76E-71 | 5.73E-69 |
| CES1 | 0.116 | 0.095 | 0.137 | 1.42E-27 | 2.62E-26 |
| GUSB | 0.116 | 0.100 | 0.131 | 6.62E-48 | 4.72E-46 |
| GSTA1 | 0.114 | 0.093 | 0.136 | 6.55E-25 | 9.82E-24 |
| ANXA10 | 0.109 | 0.09 | 0.128 | 2.84E-28 | 5.54E-27 |
| TREM2 | 0.106 | 0.093 | 0.119 | 6.53E-58 | 9.09E-56 |
| ACE2 | 0.105 | 0.092 | 0.118 | 3.69E-60 | 6.75E-58 |
| RARRES2 | 0.103 | 0.09 | 0.115 | 8.04E-58 | 1.07E-55 |
| ADH4 | 0.102 | 0.081 | 0.123 | 5.54E-21 | 6.30E-20 |
| CCL19 | 0.101 | 0.071 | 0.131 | 4.37E-11 | 2.44E-10 |
| CHI3L1 | 0.101 | 0.082 | 0.119 | 1.57E-25 | 2.50E-24 |
| MME | 0.101 | 0.081 | 0.12 | 1.01E-23 | 1.40E-22 |
| VSIG2 | 0.101 | 0.088 | 0.113 | 1.21E-55 | 1.48E-53 |
| ADGRG1 | 0.099 | 0.082 | 0.116 | 2.51E-29 | 5.21E-28 |
| COL6A3 | 0.097 | 0.088 | 0.106 | 3.23E-98 | 4.72E-95 |
| IGSF9 | 0.097 | 0.077 | 0.116 | 1.55E-22 | 1.94E-21 |
| CCL20 | 0.096 | 0.075 | 0.118 | 4.18E-18 | 3.92E-17 |
| CCL7 | 0.096 | 0.082 | 0.111 | 7.90E-38 | 2.82E-36 |
| HAVCR1 | 0.096 | 0.081 | 0.112 | 4.09E-34 | 1.06E-32 |
| DEFB4A | 0.093 | 0.058 | 0.129 | 2.28E-07 | 9.14E-07 |
| DPY30 | 0.091 | 0.076 | 0.106 | 1.97E-32 | 4.67E-31 |
| TFF1 | 0.091 | 0.072 | 0.11 | 1.67E-20 | 1.85E-19 |
| IGFBP4 | 0.089 | 0.078 | 0.099 | 4.65E-60 | 8.00E-58 |
| MSR1 | 0.088 | 0.077 | 0.100 | 7.75E-51 | 6.66E-49 |
| GDF15 | 0.087 | 0.076 | 0.098 | 1.20E-52 | 1.21E-50 |
| ALDH3A1 | 0.086 | 0.07 | 0.102 | 4.69E-25 | 7.17E-24 |
| FCAMR | 0.086 | 0.07 | 0.102 | 1.99E-25 | 3.13E-24 |
| RBP5 | 0.085 | 0.071 | 0.100 | 1.10E-30 | 2.45E-29 |
| AGR2 | 0.084 | 0.061 | 0.108 | 2.52E-12 | 1.56E-11 |
| LGALS4 | 0.084 | 0.071 | 0.098 | 3.92E-35 | 1.10E-33 |
| PLAT | 0.084 | 0.071 | 0.098 | 3.81E-36 | 1.16E-34 |
| MMP12 | 0.083 | 0.07 | 0.097 | 3.06E-35 | 8.76E-34 |
| SIT1 | 0.083 | 0.069 | 0.097 | 1.05E-32 | 2.54E-31 |
| SKAP1 | 0.083 | 0.066 | 0.102 | 6.18E-20 | 6.49E-19 |
| FUOM | 0.083 | 0.069 | 0.096 | 1.62E-32 | 3.88E-31 |
| DNER | -0.040 | -0.046 | -0.033 | 1.10E-33 | 2.74E-32 |
| CRH | -0.041 | -0.062 | -0.020 | 1.09E-04 | 3.07E-04 |
| APLP1 | -0.042 | -0.054 | -0.029 | 1.72E-10 | 9.04E-10 |
| ENPP6 | -0.042 | -0.05 | -0.034 | 1.23E-25 | 1.98E-24 |
| SCT | -0.042 | -0.051 | -0.033 | 2.41E-18 | 2.30E-17 |
| CKB | -0.043 | -0.057 | -0.03 | 7.22E-10 | 3.63E-09 |
| DPP6 | -0.043 | -0.052 | -0.035 | 2.87E-24 | 4.16E-23 |
| HBZ | -0.043 | -0.065 | -0.021 | 0.0001513 | 0.0004205 |
| ICAM4 | -0.044 | -0.057 | -0.032 | 7.40E-12 | 4.48E-11 |
| LRRN1 | -0.044 | -0.053 | -0.035 | 6.28E-20 | 6.58E-19 |
| ODAM | -0.044 | -0.057 | -0.031 | 1.83E-11 | 1.07E-10 |
| RAB6A | -0.044 | -0.056 | -0.033 | 3.03E-14 | 2.15E-13 |
| CTRC | -0.045 | -0.06 | -0.03 | 9.19E-09 | 4.21E-08 |
| LPO | -0.045 | -0.059 | -0.03 | 1.65E-09 | 8.01E-09 |
| CTSB | -0.046 | -0.06 | -0.034 | 2.32E-12 | 1.45E-11 |
| LEG1 | -0.046 | -0.06 | -0.032 | 8.17E-11 | 4.46E-10 |
| PLA2G1B | -0.046 | -0.056 | -0.035 | 5.34E-17 | 4.69E-16 |
| WFIKKN2 | -0.046 | -0.054 | -0.037 | 6.98E-25 | 1.04E-23 |
| OBP2B | -0.047 | -0.063 | -0.032 | 8.55E-10 | 4.28E-09 |
| NELL1 | -0.048 | -0.058 | -0.038 | 3.99E-20 | 4.29E-19 |
| AFP | -0.049 | -0.065 | -0.033 | 2.04E-09 | 9.83E-09 |
| AMY2B | -0.049 | -0.061 | -0.038 | 9.65E-18 | 8.87E-17 |
| BCAN | -0.049 | -0.058 | -0.041 | 1.86E-29 | 3.88E-28 |
| IGFBP2 | -0.049 | -0.066 | -0.032 | 1.21E-08 | 5.50E-08 |
| PLTP | -0.049 | -0.058 | -0.04 | 8.30E-27 | 1.46E-25 |
| PON3 | -0.049 | -0.057 | -0.041 | 6.88E-36 | 2.05E-34 |
| IL1RAP | -0.050 | -0.062 | -0.04 | 6.90E-20 | 7.20E-19 |
| AMY2A | -0.051 | -0.063 | -0.04 | 8.40E-19 | 8.13E-18 |
| CA14 | -0.052 | -0.061 | -0.043 | 1.31E-29 | 2.75E-28 |
| NCAN | -0.052 | -0.062 | -0.043 | 3.44E-26 | 5.78E-25 |
| SPINT3 | -0.054 | -0.092 | -0.016 | 0.0049916 | 0.010304 |
| GAL | -0.056 | -0.071 | -0.039 | 1.49E-11 | 8.78E-11 |
| ADAMTS8 | -0.058 | -0.068 | -0.046 | 4.02E-24 | 5.76E-23 |
| CELA3A | -0.058 | -0.071 | -0.043 | 7.72E-16 | 6.28E-15 |
| CRISP2 | -0.060 | -0.074 | -0.046 | 1.21E-16 | 1.05E-15 |
| SHBG | -0.060 | -0.071 | -0.048 | 1.19E-23 | 1.63E-22 |
| BPIFB1 | -0.064 | -0.080 | -0.048 | 5.67E-15 | 4.31E-14 |
| CST1 | -0.065 | -0.084 | -0.046 | 3.16E-11 | 1.79E-10 |
| OMG | -0.067 | -0.084 | -0.051 | 5.81E-16 | 4.77E-15 |
| KLK1 | -0.070 | -0.101 | -0.040 | 5.922E-06 | 1.992E-05 |
| KLK3 | -0.070 | -0.092 | -0.049 | 1.79E-10 | 9.39E-10 |
| PPY | -0.071 | -0.096 | -0.047 | 1.66E-08 | 7.492E-08 |
| CELA2A | -0.076 | -0.088 | -0.063 | 4.29E-31 | 9.80E-30 |
| CA6 | -0.078 | -0.094 | -0.063 | 4.19E-23 | 5.44E-22 |
| UMOD | -0.079 | -0.093 | -0.066 | 1.48E-31 | 3.47E-30 |
| XPNPEP2 | -0.100 | -0.123 | -0.076 | 2.53E-16 | 2.12E-15 |
| IGFBP1 | -0.106 | -0.139 | -0.074 | 1.29E-10 | 6.92E-10 |
| GP2 | -0.111 | -0.131 | -0.091 | 4.88E-27 | 8.76E-26 |
| GH1 | -0.129 | -0.173 | -0.085 | 6.63E-09 | 3.06E-08 |
| BPIFA2 | -0.130 | -0.152 | -0.107 | 6.60E-30 | 1.42E-28 |

**Abbreviations:** FDR: False discovery rate; HR: Hazard ratio; CI: Confidence interval.

**Supplement Table 18. GO, KEGG and Reactome enrichment analysis of DJD-associated proteins**

| **ID** | **ONTOLOGY** | **Description** | **pvalue** | **Count** | **p.adjust** |
| --- | --- | --- | --- | --- | --- |
| GO:0002684 | BP | positive regulation of immune system process | 3.02E-06 | 239 | 0.005799 |
| GO:0009617 | BP | response to bacterium | 3.45E-06 | 176 | 0.005799 |
| GO:0032101 | BP | regulation of response to external stimulus | 8.51E-06 | 261 | 0.0095182 |
| GO:0046718 | BP | viral entry into host cell | 1.49E-05 | 62 | 0.0125159 |
| GO:0002687 | BP | positive regulation of leukocyte migration | 3.41E-05 | 62 | 0.0164793 |
| GO:0009607 | BP | response to biotic stimulus | 3.59E-05 | 333 | 0.0164793 |
| GO:0050776 | BP | regulation of immune response | 4.42E-05 | 209 | 0.0164793 |
| GO:0000165 | BP | MAPK cascade | 5.21E-05 | 188 | 0.0164793 |
| GO:0044000 | BP | movement in host | 6.01E-05 | 65 | 0.0164793 |
| GO:0002685 | BP | regulation of leukocyte migration | 6.33E-05 | 85 | 0.0164793 |
| GO:0002683 | BP | negative regulation of immune system process | 6.53E-05 | 128 | 0.0164793 |
| GO:0016477 | BP | cell migration | 7.27E-05 | 337 | 0.0164793 |
| GO:0002688 | BP | regulation of leukocyte chemotaxis | 8.20E-05 | 52 | 0.0164793 |
| GO:0019058 | BP | viral life cycle | 8.35E-05 | 84 | 0.0164793 |
| GO:0022409 | BP | positive regulation of cell-cell adhesion | 1.30E-04 | 97 | 0.0217471 |
| GO:0030194 | BP | positive regulation of blood coagulation | 1.55E-04 | 17 | 0.0217471 |
| GO:0050820 | BP | positive regulation of coagulation | 1.55E-04 | 17 | 0.0217471 |
| GO:1900048 | BP | positive regulation of hemostasis | 1.55E-04 | 17 | 0.0217471 |
| GO:0051701 | BP | biological process involved in interaction with host | 1.89E-04 | 68 | 0.0249214 |
| GO:0050865 | BP | regulation of cell activation | 1.93E-04 | 183 | 0.0249214 |
| GO:0008285 | BP | negative regulation of cell population proliferation | 2.08E-04 | 168 | 0.0258618 |
| GO:0001890 | BP | placenta development | 0.0003101 | 38 | 0.0346956 |
| GO:0051241 | BP | negative regulation of multicellular organismal process | 0.0003493 | 238 | 0.0368757 |
| GO:0009611 | BP | response to wounding | 0.0003845 | 172 | 0.0368757 |
| GO:0018108 | BP | peptidyl-tyrosine phosphorylation | 0.0004946 | 115 | 0.0393539 |
| GO:0018212 | BP | peptidyl-tyrosine modification | 0.0004946 | 115 | 0.0393539 |
| GO:0006954 | BP | inflammatory response | 0.0005095 | 220 | 0.0393539 |
| GO:0060326 | BP | cell chemotaxis | 0.0005263 | 107 | 0.0393539 |
| GO:0051251 | BP | positive regulation of lymphocyte activation | 0.0005566 | 95 | 0.0393539 |
| GO:0009986 | CC | cell surface | 2.826E-06 | 295 | 0.0010342 |
| GO:0009897 | CC | external side of plasma membrane | 3.548E-05 | 151 | 0.0052153 |
| GO:0030141 | CC | secretory granule | 6.379E-05 | 234 | 0.0052153 |
| GO:0030312 | CC | external encapsulating structure | 7.125E-05 | 185 | 0.0052153 |
| GO:0031012 | CC | extracellular matrix | 7.125E-05 | 185 | 0.0052153 |
| GO:0098552 | CC | side of membrane | 0.0003117 | 179 | 0.0138381 |
| GO:0005783 | CC | endoplasmic reticulum | 0.0005304 | 254 | 0.0194115 |
| GO:0043202 | CC | lysosomal lumen | 0.0010917 | 46 | 0.0363239 |
| GO:0098857 | CC | membrane microdomain | 0.0018805 | 85 | 0.0434722 |
| GO:0045121 | CC | membrane raft | 0.0023411 | 84 | 0.0434722 |
| hsa04610 | KEGG | Complement and coagulation cascades | 0.0002858 | 49 | 0.0452327 |
| hsa04142 | KEGG | Lysosome | 0.0003534 | 43 | 0.0452327 |
| hsa05171 | KEGG | Coronavirus disease - COVID-19 | 0.0013098 | 49 | 0.111773 |
| hsa04148 | KEGG | Efferocytosis | 0.0097402 | 37 | 0.6233705 |
| hsa00600 | KEGG | Sphingolipid metabolism | 0.0170963 | 12 | 0.6732924 |
| hsa04612 | KEGG | Antigen processing and presentation | 0.0173788 | 23 | 0.6732924 |
| hsa05133 | KEGG | Pertussis | 0.0184103 | 30 | 0.6732924 |
| hsa05144 | KEGG | Malaria | 0.0221425 | 27 | 0.7085591 |
| hsa00564 | KEGG | Glycerophospholipid metabolism | 0.0364705 | 13 | 0.8387378 |
| hsa05150 | KEGG | Staphylococcus aureus infection | 0.0381139 | 25 | 0.8387378 |
| hsa05130 | KEGG | Pathogenic Escherichia coli infection | 0.0389566 | 42 | 0.8387378 |
| hsa04640 | KEGG | Hematopoietic cell lineage | 3.93E-02 | 44 | 8.39E-01 |
| hsa04620 | KEGG | Toll-like receptor signaling pathway | 4.39E-02 | 31 | 8.65E-01 |
| R-HSA-6798695 | Reactome | Neutrophil degranulation | 4.67E-06 | 144 | 2.61E-03 |
| R-HSA-168249 | Reactome | Innate Immune System | 9.23E-05 | 244 | 0.0248927 |
| R-HSA-166658 | Reactome | Complement cascade | 1.34E-04 | 29 | 0.0248927 |
| R-HSA-977606 | Reactome | Regulation of Complement cascade | 1.78E-04 | 25 | 0.0248927 |
| R-HSA-5663084 | Reactome | Diseases of carbohydrate metabolism | 0.0036911 | 11 | 0.4119306 |
| R-HSA-6783783 | Reactome | Interleukin-10 signaling | 0.0047828 | 28 | 0.4448016 |
| R-HSA-114608 | Reactome | Platelet degranulation | 0.0110393 | 49 | 0.7762184 |
| R-HSA-5669034 | Reactome | TNFs bind their physiological receptors | 0.0112058 | 18 | 0.7762184 |
| R-HSA-381426 | Reactome | Regulation of Insulin-like Growth Factor (IGF) transport and uptake by Insulin-like Growth Factor Binding Proteins (IGFBPs) | 1.37E-02 | 54 | 0.7762184 |
| R-HSA-76005 | Reactome | Response to elevated platelet cytosolic Ca2+ | 0.0139107 | 50 | 0.7762184 |
| R-HSA-8957275 | Reactome | Post-translational protein phosphorylation | 0.0175461 | 47 | 0.8173226 |
| R-HSA-375276 | Reactome | Peptide ligand-binding receptors | 0.0176183 | 45 | 0.8173226 |
| R-HSA-166663 | Reactome | Initial triggering of complement | 0.0199398 | 11 | 0.8173226 |
| R-HSA-198933 | Reactome | Immunoregulatory interactions between a Lymphoid and a non-Lymphoid cell | 0.0232027 | 61 | 0.8173226 |
| R-HSA-449836 | Reactome | Other interleukin signaling | 0.0233805 | 16 | 0.8173226 |
| R-HSA-556833 | Reactome | Metabolism of lipids | 0.0245535 | 88 | 0.8173226 |
| R-HSA-73887 | Reactome | Death Receptor Signalling | 0.0271784 | 29 | 0.8173226 |
| R-HSA-5668914 | Reactome | Diseases of metabolism | 0.029613 | 56 | 0.8173226 |
| R-HSA-163560 | Reactome | Triglyceride catabolism | 0.0307595 | 10 | 0.8173226 |
| R-HSA-75205 | Reactome | Dissolution of Fibrin Clot | 0.0307595 | 10 | 0.8173226 |
| R-HSA-8979227 | Reactome | Triglyceride metabolism | 0.0307595 | 10 | 0.8173226 |

**Abbreviations:** BP: Biological process; CC: Cellular component; MF: Molecular function; KEGG: Kyoto Encyclopedia of Genes and Genomes.

**Supplement Table 19. Identification of top OA-associated proteins using generalized linear regression**

| **Proteins** | **Beta** | **CI_lower** | **CI_upper** | **pvalue** | **FDR** |
| --- | --- | --- | --- | --- | --- |
| GAST | 0.293 | 0.26 | 0.324 | 1.40E-71 | 2.41E-69 |
| LEP | 0.289 | 0.261 | 0.317 | 5.71E-91 | 2.384E-88 |
| COL9A1 | 0.225 | 0.211 | 0.237 | 6.28E-247 | 1.83E-243 |
| FABP4 | 0.196 | 0.177 | 0.213 | 5.66E-102 | 4.13E-99 |
| SSC4D | 0.195 | 0.16 | 0.23 | 3.68E-27 | 4.622E-26 |
| CHGA | 0.175 | 0.153 | 0.196 | 1.17E-58 | 9.49E-57 |
| IL1RN | 0.166 | 0.149 | 0.184 | 1.04E-77 | 2.532E-75 |
| FABP1 | 0.165 | 0.137 | 0.193 | 4.38E-31 | 7.23E-30 |
| FGF21 | 0.164 | 0.128 | 0.199 | 3.061E-19 | 2.438E-18 |
| KRT18 | 0.163 | 0.139 | 0.187 | 6.554E-40 | 1.583E-38 |
| IL6 | 0.160 | 0.139 | 0.183 | 2.22E-46 | 7.83E-45 |
| OXT | 0.159 | 0.117 | 0.201 | 2.083E-13 | 1.182E-12 |
| CES1 | 0.151 | 0.127 | 0.176 | 7.249E-34 | 1.422E-32 |
| CDCP1 | 0.150 | 0.136 | 0.165 | 2.252E-87 | 8.229E-85 |
| GUSB | 0.150 | 0.132 | 0.168 | 4.304E-58 | 3.145E-56 |
| GSTA1 | 0.144 | 0.119 | 0.17 | 1.852E-28 | 2.585E-27 |
| ANXA10 | 0.141 | 0.118 | 0.163 | 7.093E-34 | 1.401E-32 |
| CCL7 | 0.141 | 0.123 | 0.157 | 4.845E-57 | 3.013E-55 |
| DEFB4A | 0.141 | 0.099 | 0.183 | 4.011E-11 | 1.909E-10 |
| RARRES2 | 0.139 | 0.124 | 0.154 | 1.438E-76 | 3.234E-74 |
| CHI3L1 | 0.134 | 0.111 | 0.156 | 2.82E-32 | 5.16E-31 |
| CCL19 | 0.130 | 0.095 | 0.165 | 4.654E-13 | 2.557E-12 |
| CCL20 | 0.130 | 0.104 | 0.155 | 2.443E-23 | 2.42E-22 |
| ACE2 | 0.129 | 0.115 | 0.144 | 1.75E-66 | 2.43E-64 |
| TREM2 | 0.128 | 0.113 | 0.144 | 1.625E-61 | 1.583E-59 |
| COL6A3 | 0.128 | 0.117 | 0.138 | 7.01E-124 | 1.02E-120 |
| IGSF9 | 0.128 | 0.105 | 0.15 | 7.349E-28 | 9.633E-27 |
| ADGRG1 | 0.127 | 0.106 | 0.146 | 9.60E-35 | 1.98E-33 |
| HAVCR1 | 0.126 | 0.107 | 0.144 | 2.32E-41 | 6.10E-40 |
| TFF1 | 0.126 | 0.103 | 0.148 | 9.56E-28 | 1.24E-26 |
| MME | 0.124 | 0.101 | 0.147 | 4.29E-26 | 5.119E-25 |
| ADH4 | 0.122 | 0.097 | 0.147 | 7.917E-22 | 7.323E-21 |
| DPY30 | 0.120 | 0.102 | 0.137 | 2.514E-40 | 6.228E-39 |
| VSIG2 | 0.117 | 0.102 | 0.131 | 3.51E-55 | 1.94E-53 |
| AGR2 | 0.115 | 0.087 | 0.142 | 4.46E-16 | 3.00E-15 |
| PLAT | 0.115 | 0.1 | 0.131 | 4.952E-48 | 1.905E-46 |
| IGFBP4 | 0.114 | 0.102 | 0.127 | 4.55E-72 | 8.32E-70 |
| MSR1 | 0.114 | 0.101 | 0.128 | 4.652E-62 | 4.856E-60 |
| FCAMR | 0.113 | 0.094 | 0.132 | 7.072E-32 | 1.261E-30 |
| KLK4 | 0.112 | 0.094 | 0.13 | 1.72E-34 | 3.46E-33 |
| RBP5 | 0.111 | 0.094 | 0.128 | 4.385E-37 | 9.494E-36 |
| SNCG | 0.111 | 0.091 | 0.13 | 4.765E-28 | 6.419E-27 |
| ADAMTS15 | 0.110 | 0.097 | 0.124 | 7.41E-58 | 5.282E-56 |
| CCL16 | 0.109 | 0.093 | 0.124 | 1.501E-42 | 4.219E-41 |
| GDF15 | 0.109 | 0.095 | 0.122 | 2.85E-59 | 2.38E-57 |
| LGALS4 | 0.107 | 0.091 | 0.123 | 5.01E-41 | 1.29E-39 |
| TMSB10 | 0.107 | 0.085 | 0.129 | 9.157E-22 | 8.417E-21 |
| CA5A | 0.104 | 0.078 | 0.13 | 6.951E-15 | 4.36E-14 |
| CCN3 | 0.104 | 0.095 | 0.114 | 2.523E-97 | 1.229E-94 |
| CSTB | 0.104 | 0.092 | 0.117 | 3.80E-60 | 3.472E-58 |
| AMY1A | -0.049 | -0.062 | -0.038 | 2.499E-16 | 1.706E-15 |
| DPP6 | -0.049 | -0.059 | -0.039 | 1.246E-22 | 1.202E-21 |
| APLP1 | -0.050 | -0.066 | -0.036 | 3.322E-11 | 1.592E-10 |
| CTSB | -0.050 | -0.066 | -0.035 | 1.039E-10 | 4.788E-10 |
| DNER | -0.050 | -0.058 | -0.042 | 3.033E-38 | 6.767E-37 |
| ODAM | -0.050 | -0.065 | -0.035 | 6.702E-11 | 3.135E-10 |
| LPO | -0.051 | -0.068 | -0.034 | 3.329E-09 | 1.333E-08 |
| RAB6A | -0.051 | -0.064 | -0.038 | 6.10E-14 | 3.60E-13 |
| CRH | -0.052 | -0.077 | -0.028 | 2.864E-05 | 7.26E-05 |
| ENPP6 | -0.052 | -0.062 | -0.043 | 1.85E-28 | 2.585E-27 |
| AFP | -0.053 | -0.073 | -0.035 | 2.426E-08 | 8.955E-08 |
| CKB | -0.053 | -0.070 | -0.038 | 8.04E-11 | 3.74E-10 |
| PLA2G1B | -0.053 | -0.066 | -0.041 | 3.743E-17 | 2.675E-16 |
| GHRL | -0.054 | -0.078 | -0.030 | 9.476E-06 | 2.576E-05 |
| LEG1 | -0.054 | -0.071 | -0.038 | 5.467E-11 | 2.573E-10 |
| SCT | -0.054 | -0.065 | -0.043 | 7.404E-22 | 6.871E-21 |
| LRRN1 | -0.057 | -0.067 | -0.045 | 2.93E-23 | 2.89E-22 |
| WFIKKN2 | -0.057 | -0.067 | -0.047 | 6.828E-28 | 8.99E-27 |
| PLTP | -0.058 | -0.068 | -0.047 | 2.512E-26 | 3.022E-25 |
| IGFBP2 | -0.059 | -0.079 | -0.039 | 8.87E-09 | 3.42E-08 |
| IL1RAP | -0.059 | -0.071 | -0.046 | 2.471E-19 | 1.989E-18 |
| SCGB3A2 | -0.059 | -0.084 | -0.035 | 2.498E-06 | 7.28E-06 |
| BCAN | -0.060 | -0.070 | -0.050 | 1.91E-31 | 3.29E-30 |
| CTRC | -0.060 | -0.078 | -0.041 | 1.078E-10 | 4.964E-10 |
| AMY2B | -0.062 | -0.075 | -0.049 | 6.855E-20 | 5.775E-19 |
| CA14 | -0.064 | -0.074 | -0.053 | 3.188E-32 | 5.716E-31 |
| NELL1 | -0.064 | -0.076 | -0.052 | 1.117E-25 | 1.301E-24 |
| OBP2B | -0.064 | -0.082 | -0.046 | 1.868E-12 | 9.874E-12 |
| PON3 | -0.064 | -0.073 | -0.055 | 2.754E-43 | 8.132E-42 |
| AMY2A | -0.065 | -0.079 | -0.052 | 8.049E-22 | 7.422E-21 |
| BPIFB1 | -0.065 | -0.084 | -0.046 | 1.047E-11 | 5.279E-11 |
| OMG | -0.065 | -0.084 | -0.046 | 2.49E-11 | 1.21E-10 |
| PPY | -0.067 | -0.097 | -0.038 | 5.322E-06 | 1.497E-05 |
| NCAN | -0.069 | -0.081 | -0.058 | 3.441E-33 | 6.49E-32 |
| CELA3A | -0.070 | -0.086 | -0.054 | 5.387E-17 | 3.831E-16 |
| CRISP2 | -0.070 | -0.087 | -0.054 | 1.024E-16 | 7.14E-16 |
| KLK1 | -0.070 | -0.106 | -0.034 | 0.0001275 | 0.0002879 |
| CST1 | -0.073 | -0.095 | -0.050 | 2.55E-10 | 1.139E-09 |
| GAL | -0.074 | -0.092 | -0.055 | 1.596E-14 | 9.844E-14 |
| ADAMTS8 | -0.075 | -0.088 | -0.062 | 1.33E-29 | 2.04E-28 |
| SHBG | -0.076 | -0.09 | -0.063 | 5.14E-28 | 6.893E-27 |
| KLK3 | -0.079 | -0.105 | -0.054 | 1.11E-09 | 4.676E-09 |
| CELA2A | -0.089 | -0.104 | -0.074 | 3.369E-31 | 5.628E-30 |
| CA6 | -0.095 | -0.113 | -0.077 | 1.84E-24 | 1.975E-23 |
| UMOD | -0.106 | -0.122 | -0.091 | 6.358E-41 | 1.616E-39 |
| IGFBP1 | -0.118 | -0.156 | -0.079 | 1.67E-09 | 6.855E-09 |
| GP2 | -0.122 | -0.146 | -0.099 | 7.123E-24 | 7.305E-23 |
| XPNPEP2 | -0.127 | -0.154 | -0.098 | 8.083E-19 | 6.234E-18 |
| GH1 | -0.155 | -0.207 | -0.105 | 2.525E-09 | 1.024E-08 |
| BPIFA2 | -0.157 | -0.183 | -0.131 | 1.435E-31 | 2.511E-30 |

**Supplement Table 20. Identification of top IVDD-associated proteins using generalized linear regression**

| **Proteins** | **Beta** | **CI_lower** | **CI_upper** | **pvalue** | **FDR** |
| --- | --- | --- | --- | --- | --- |
| GAST | 0.199 | 0.145 | 0.253 | 6.389E-13 | 1.698E-10 |
| MYOM3 | 0.182 | 0.142 | 0.222 | 4.108E-19 | 4.003E-16 |
| LEP | 0.152 | 0.103 | 0.201 | 1.4E-09 | 1.279E-07 |
| CHGA | 0.135 | 0.100 | 0.171 | 7.723E-14 | 3.225E-11 |
| CSRP3 | 0.123 | 0.083 | 0.164 | 2.353E-09 | 1.965E-07 |
| MYBPC1 | 0.119 | 0.088 | 0.149 | 3.141E-14 | 1.53E-11 |
| ALDH3A1 | 0.114 | 0.081 | 0.147 | 1.258E-11 | 1.935E-09 |
| ADH4 | 0.112 | 0.068 | 0.156 | 5.453E-07 | 1.853E-05 |
| MYL3 | 0.102 | 0.070 | 0.132 | 1.786E-10 | 1.8E-08 |
| ADH1B | 0.101 | 0.067 | 0.134 | 3.408E-09 | 2.692E-07 |
| FABP4 | 0.101 | 0.069 | 0.131 | 2.566E-10 | 2.5E-08 |
| KRT18 | 0.099 | 0.057 | 0.141 | 4.462E-06 | 0.0001011 |
| GSTA1 | 0.098 | 0.053 | 0.143 | 1.915E-05 | 0.0003611 |
| HSPB6 | 0.097 | 0.077 | 0.117 | 5.458E-22 | 1.595E-18 |
| ALPP | 0.096 | 0.035 | 0.158 | 0.0021258 | 0.0175032 |
| HAO1 | 0.096 | 0.031 | 0.162 | 0.0039911 | 0.0284535 |
| MME | 0.096 | 0.056 | 0.136 | 3.016E-06 | 7.053E-05 |
| CES1 | 0.095 | 0.053 | 0.138 | 1.044E-05 | 0.0002133 |
| VSIG2 | 0.094 | 0.07 | 0.12 | 9.968E-14 | 3.642E-11 |
| MB | 0.093 | 0.066 | 0.119 | 9.606E-12 | 1.56E-09 |
| COL9A1 | 0.091 | 0.069 | 0.113 | 1.044E-15 | 7.629E-13 |
| FUOM | 0.091 | 0.063 | 0.119 | 1.627E-10 | 1.698E-08 |
| ACE2 | 0.090 | 0.064 | 0.115 | 4.194E-12 | 7.661E-10 |
| FTCD | 0.086 | 0.047 | 0.125 | 1.267E-05 | 0.0002536 |
| TREM2 | 0.084 | 0.058 | 0.111 | 4.025E-10 | 3.795E-08 |
| IL1RN | 0.083 | 0.053 | 0.114 | 7.472E-08 | 4.121E-06 |
| PGA4 | 0.083 | 0.053 | 0.112 | 4.735E-08 | 2.714E-06 |
| TSPAN8 | 0.083 | 0.04 | 0.125 | 0.0001418 | 0.0019453 |
| UPB1 | 0.082 | 0.04 | 0.123 | 0.0001157 | 0.0016412 |
| FABP1 | 0.081 | 0.032 | 0.130 | 0.0011811 | 0.0108909 |
| RBFOX3 | 0.081 | 0.064 | 0.098 | 1.305E-20 | 1.907E-17 |
| AGR2 | 0.080 | 0.031 | 0.128 | 0.0012897 | 0.0117435 |
| THBS4 | 0.080 | 0.056 | 0.103 | 2.201E-11 | 3.217E-09 |
| CAPG | 0.079 | 0.049 | 0.109 | 1.793E-07 | 7.823E-06 |
| GSTA3 | 0.076 | 0.036 | 0.115 | 0.0001572 | 0.0021082 |
| TCL1A | 0.076 | 0.026 | 0.126 | 0.0028157 | 0.0220064 |
| HAVCR1 | 0.074 | 0.043 | 0.106 | 4.613E-06 | 0.0001037 |
| COL6A3 | 0.072 | 0.054 | 0.091 | 8.705E-15 | 5.089E-12 |
| DTNB | 0.072 | 0.053 | 0.092 | 2.223E-13 | 6.497E-11 |
| ACAA1 | 0.070 | 0.030 | 0.110 | 0.000683 | 0.0070543 |
| LGALS4 | 0.070 | 0.043 | 0.098 | 3.547E-07 | 1.364E-05 |
| NOS1 | 0.070 | 0.044 | 0.097 | 1.435E-07 | 6.658E-06 |
| EDA2R | 0.070 | 0.050 | 0.088 | 9.811E-13 | 2.374E-10 |
| ANXA10 | 0.069 | 0.029 | 0.108 | 0.0006437 | 0.0067198 |
| PTN | 0.069 | 0.045 | 0.093 | 3.014E-08 | 1.855E-06 |
| SIT1 | 0.069 | 0.041 | 0.097 | 1.468E-06 | 4.009E-05 |
| ACTN2 | 0.067 | 0.042 | 0.092 | 1.382E-07 | 6.547E-06 |
| CA3 | 0.067 | 0.036 | 0.098 | 2.621E-05 | 0.0004806 |
| GPD1 | 0.067 | 0.042 | 0.092 | 8.534E-08 | 4.535E-06 |
| BST2 | 0.066 | 0.039 | 0.092 | 1.605E-06 | 4.253E-05 |
| APOM | -0.035 | -0.049 | -0.021 | 1.337E-06 | 3.83E-05 |
| ITGA11 | -0.035 | -0.052 | -0.017 | 0.0001039 | 0.0015108 |
| AMY2A | -0.036 | -0.058 | -0.012 | 0.003182 | 0.0239096 |
| APLP1 | -0.036 | -0.062 | -0.010 | 0.0072767 | 0.0460387 |
| CEND1 | -0.036 | -0.06 | -0.011 | 0.0045816 | 0.0316599 |
| PLTP | -0.036 | -0.054 | -0.017 | 0.0001946 | 0.0024629 |
| NPTXR | -0.037 | -0.054 | -0.020 | 2.439E-05 | 0.0004513 |
| CD209 | -0.038 | -0.058 | -0.019 | 0.0001046 | 0.0015134 |
| CHRDL2 | -0.038 | -0.065 | -0.010 | 0.0067665 | 0.0436891 |
| ODAM | -0.038 | -0.064 | -0.011 | 0.0050303 | 0.0341153 |
| AGRP | -0.039 | -0.066 | -0.012 | 0.0044871 | 0.031154 |
| ITGAM | -0.039 | -0.054 | -0.022 | 2.548E-06 | 6.207E-05 |
| BCAN | -0.040 | -0.057 | -0.022 | 8.738E-06 | 0.0001851 |
| APOD | -0.041 | -0.059 | -0.023 | 1.013E-05 | 0.0002086 |
| KITLG | -0.041 | -0.06 | -0.022 | 1.721E-05 | 0.0003288 |
| PNLIP | -0.041 | -0.067 | -0.014 | 0.0031342 | 0.0236112 |
| CTSB | -0.042 | -0.069 | -0.016 | 0.0019174 | 0.0161516 |
| CKB | -0.043 | -0.072 | -0.015 | 0.0028932 | 0.0223346 |
| CNTN2 | -0.043 | -0.066 | -0.021 | 0.0001556 | 0.0020959 |
| CRISP2 | -0.043 | -0.072 | -0.014 | 0.0038867 | 0.0279821 |
| GRP | -0.043 | -0.065 | -0.020 | 0.0001887 | 0.0024085 |
| HPGDS | -0.043 | -0.062 | -0.025 | 3.461E-06 | 8.028E-05 |
| CCL28 | -0.045 | -0.074 | -0.015 | 0.0028094 | 0.0220064 |
| DMP1 | -0.045 | -0.071 | -0.019 | 0.0007121 | 0.0073292 |
| NCAN | -0.045 | -0.065 | -0.025 | 9.388E-06 | 0.000196 |
| PLA2G1B | -0.045 | -0.067 | -0.024 | 4.323E-05 | 0.0007292 |
| IL1RAP | -0.046 | -0.068 | -0.024 | 6.002E-05 | 0.0009692 |
| CST5 | -0.047 | -0.075 | -0.020 | 0.000728 | 0.0074166 |
| DPP6 | -0.048 | -0.065 | -0.031 | 4.623E-08 | 2.714E-06 |
| SHBG | -0.048 | -0.072 | -0.025 | 6.773E-05 | 0.0010587 |
| CHGB | -0.050 | -0.071 | -0.029 | 2.302E-06 | 5.751E-05 |
| OPTC | -0.050 | -0.073 | -0.028 | 1.379E-05 | 0.0002705 |
| HSD11B1 | -0.051 | -0.072 | -0.031 | 1.035E-06 | 3.252E-05 |
| ICAM4 | -0.051 | -0.077 | -0.025 | 0.0001391 | 0.0019265 |
| LEFTY2 | -0.054 | -0.081 | -0.028 | 5.232E-05 | 0.0008689 |
| AFP | -0.056 | -0.088 | -0.022 | 0.00117 | 0.0108566 |
| LPO | -0.059 | -0.089 | -0.029 | 0.0001301 | 0.0018202 |
| CELA3A | -0.060 | -0.088 | -0.031 | 4.341E-05 | 0.0007292 |
| CPA2 | -0.060 | -0.093 | -0.027 | 0.0003176 | 0.0038525 |
| CST1 | -0.064 | -0.103 | -0.025 | 0.001459 | 0.0128451 |
| CA6 | -0.066 | -0.099 | -0.035 | 4.649E-05 | 0.0007765 |
| POMC | -0.074 | -0.104 | -0.044 | 1.506E-06 | 4.05E-05 |
| CELA2A | -0.086 | -0.112 | -0.059 | 1.238E-10 | 1.391E-08 |
| OMG | -0.088 | -0.121 | -0.054 | 3.412E-07 | 1.33E-05 |
| BPIFB1 | -0.089 | -0.122 | -0.056 | 1.106E-07 | 5.389E-06 |
| BPIFA2 | -0.094 | -0.140 | -0.048 | 6.245E-05 | 0.0010024 |
| IGFBP1 | -0.097 | -0.163 | -0.029 | 0.0051839 | 0.035005 |
| XPNPEP2 | -0.104 | -0.154 | -0.055 | 3.249E-05 | 0.0005721 |
| GP2 | -0.139 | -0.181 | -0.098 | 5.672E-11 | 6.909E-09 |
| PPY | -0.172 | -0.222 | -0.121 | 4.189E-11 | 5.566E-09 |

**Supplement Table 21. Identification of top DSD-associated proteins using generalized linear regression**

| **Proteins** | **Beta** | **CI_lower** | **CI_upper** | **pvalue** | **FDR** |
| --- | --- | --- | --- | --- | --- |
| GAST | 0.316 | 0.271 | 0.361 | 5.163E-43 | 1.509E-39 |
| CHGA | 0.204 | 0.174 | 0.233 | 1.751E-41 | 2.559E-38 |
| LEP | 0.173 | 0.132 | 0.213 | 4.329E-17 | 3.954E-15 |
| ANXA10 | 0.138 | 0.106 | 0.170 | 5.704E-17 | 4.904E-15 |
| VSIG2 | 0.126 | 0.105 | 0.147 | 4.27E-33 | 4.16E-30 |
| ALPP | 0.122 | 0.072 | 0.172 | 2.004E-06 | 2.006E-05 |
| FABP4 | 0.119 | 0.093 | 0.144 | 7.352E-20 | 1.194E-17 |
| CDCP1 | 0.113 | 0.092 | 0.134 | 1.077E-25 | 5.247E-23 |
| TREM2 | 0.112 | 0.09 | 0.133 | 1.019E-23 | 3.308E-21 |
| CHIT1 | 0.111 | 0.048 | 0.173 | 0.0005277 | 0.0030847 |
| COL9A1 | 0.110 | 0.092 | 0.128 | 3.549E-32 | 2.593E-29 |
| REN | 0.105 | 0.074 | 0.135 | 1.589E-11 | 4.839E-10 |
| FGF21 | 0.104 | 0.053 | 0.156 | 6.242E-05 | 0.0004584 |
| KRT18 | 0.104 | 0.07 | 0.139 | 3.133E-09 | 5.985E-08 |
| AGR2 | 0.103 | 0.063 | 0.142 | 3.935E-07 | 4.753E-06 |
| ACE2 | 0.101 | 0.08 | 0.121 | 2.113E-21 | 4.752E-19 |
| LAMP3 | 0.100 | 0.077 | 0.124 | 2.803E-17 | 2.837E-15 |
| GUSB | 0.098 | 0.072 | 0.124 | 1.557E-13 | 6.895E-12 |
| GDF15 | 0.096 | 0.078 | 0.115 | 2.931E-24 | 1.071E-21 |
| CCL20 | 0.090 | 0.054 | 0.127 | 1.173E-06 | 1.242E-05 |
| FABP1 | 0.090 | 0.050 | 0.130 | 1.042E-05 | 8.881E-05 |
| CHI3L1 | 0.089 | 0.057 | 0.12 | 3.576E-08 | 5.444E-07 |
| CXADR | 0.089 | 0.068 | 0.109 | 4.964E-17 | 4.397E-15 |
| MMP12 | 0.088 | 0.066 | 0.11 | 5.541E-15 | 3.374E-13 |
| IL1RN | 0.087 | 0.062 | 0.112 | 5.91E-12 | 2.081E-10 |
| TNFRSF10B | 0.087 | 0.071 | 0.104 | 5.545E-25 | 2.316E-22 |
| MICB_MICA | 0.086 | 0.026 | 0.147 | 0.0052646 | 0.0230367 |
| SIT1 | 0.086 | 0.063 | 0.109 | 2.802E-13 | 1.187E-11 |
| GPA33 | 0.085 | 0.031 | 0.14 | 0.0020573 | 0.0102269 |
| TFF1 | 0.084 | 0.052 | 0.116 | 3.49E-07 | 4.286E-06 |
| HAVCR1 | 0.083 | 0.057 | 0.109 | 3.214E-10 | 7.517E-09 |
| PGA4 | 0.083 | 0.059 | 0.108 | 3.053E-11 | 8.582E-10 |
| ALDH3A1 | 0.083 | 0.055 | 0.109 | 2.184E-09 | 4.342E-08 |
| BST2 | 0.082 | 0.059 | 0.103 | 3.196E-13 | 1.298E-11 |
| IGFBP4 | 0.081 | 0.063 | 0.098 | 3.257E-19 | 4.76E-17 |
| MSR1 | 0.081 | 0.061 | 0.100 | 3.673E-16 | 2.684E-14 |
| LGALS4 | 0.079 | 0.056 | 0.101 | 5.199E-12 | 1.853E-10 |
| RARRES2 | 0.079 | 0.057 | 0.100 | 3.16E-13 | 1.298E-11 |
| PRSS8 | 0.078 | 0.06 | 0.096 | 8.586E-18 | 1.091E-15 |
| IL6 | 0.077 | 0.045 | 0.108 | 1.759E-06 | 1.792E-05 |
| AGR3 | 0.076 | 0.053 | 0.100 | 3.278E-10 | 7.604E-09 |
| COL6A3 | 0.076 | 0.061 | 0.091 | 2.108E-23 | 6.161E-21 |
| PIGR | 0.075 | 0.06 | 0.091 | 5.026E-21 | 1.049E-18 |
| ASGR1 | 0.073 | 0.06 | 0.086 | 7.855E-29 | 4.592E-26 |
| CCL19 | 0.073 | 0.024 | 0.124 | 0.0038051 | 0.0176543 |
| LILRB4 | 0.073 | 0.055 | 0.091 | 1.251E-15 | 8.125E-14 |
| MME | 0.073 | 0.04 | 0.106 | 1.266E-05 | 0.000106 |
| SKAP1 | 0.073 | 0.043 | 0.103 | 2.322E-06 | 2.285E-05 |
| TSPAN8 | 0.073 | 0.038 | 0.108 | 4.24E-05 | 0.0003219 |
| BGLAP | 0.072 | 0.04 | 0.105 | 9.906E-06 | 8.592E-05 |
| TNR | -0.040 | -0.057 | -0.022 | 8.931E-06 | 7.793E-05 |
| CA14 | -0.041 | -0.056 | -0.026 | 1.18E-07 | 1.597E-06 |
| DMP1 | -0.041 | -0.063 | -0.02 | 0.0001472 | 0.0010097 |
| ITGA11 | -0.041 | -0.055 | -0.027 | 1.808E-08 | 2.969E-07 |
| BCAN | -0.042 | -0.056 | -0.027 | 1.208E-08 | 2.077E-07 |
| IL7R | -0.042 | -0.063 | -0.02 | 0.0002062 | 0.0013579 |
| CNTN5 | -0.043 | -0.059 | -0.028 | 2.882E-08 | 4.458E-07 |
| BPIFB1 | -0.044 | -0.07 | -0.017 | 0.001535 | 0.0079554 |
| LEG1 | -0.044 | -0.068 | -0.021 | 0.0002063 | 0.0013579 |
| CTRB1 | -0.045 | -0.068 | -0.023 | 7.033E-05 | 0.0005101 |
| PLA2G1B | -0.045 | -0.063 | -0.027 | 7.004E-07 | 7.843E-06 |
| CPB1 | -0.046 | -0.07 | -0.022 | 0.000205 | 0.0013555 |
| NELL1 | -0.046 | -0.063 | -0.029 | 1.311E-07 | 1.757E-06 |
| NT5C3A | -0.046 | -0.082 | -0.011 | 0.0105223 | 0.0417324 |
| NUDT2 | -0.046 | -0.07 | -0.023 | 8.322E-05 | 0.0005991 |
| PLTP | -0.046 | -0.062 | -0.031 | 1.669E-09 | 3.412E-08 |
| RRM2B | -0.046 | -0.073 | -0.019 | 0.0007322 | 0.0041316 |
| CLPS | -0.047 | -0.076 | -0.019 | 0.0011199 | 0.0059844 |
| DPP6 | -0.047 | -0.061 | -0.033 | 5.641E-11 | 1.527E-09 |
| LRRN1 | -0.047 | -0.063 | -0.031 | 8.07E-09 | 1.438E-07 |
| SCT | -0.047 | -0.063 | -0.031 | 5.904E-09 | 1.079E-07 |
| HPGDS | -0.048 | -0.063 | -0.033 | 4.662E-10 | 1.032E-08 |
| IL1RAP | -0.048 | -0.067 | -0.03 | 2.617E-07 | 3.269E-06 |
| AMY2B | -0.049 | -0.069 | -0.031 | 2.859E-07 | 3.556E-06 |
| AMY2A | -0.050 | -0.07 | -0.031 | 2.359E-07 | 2.998E-06 |
| ODAM | -0.050 | -0.072 | -0.029 | 5.299E-06 | 4.81E-05 |
| POMC | -0.052 | -0.077 | -0.028 | 2.286E-05 | 0.000182 |
| CTRC | -0.054 | -0.081 | -0.029 | 3.162E-05 | 0.0002465 |
| LPO | -0.054 | -0.079 | -0.030 | 1.327E-05 | 0.0001108 |
| ICAM4 | -0.056 | -0.077 | -0.034 | 4.391E-07 | 5.197E-06 |
| PNLIP | -0.057 | -0.078 | -0.034 | 4.542E-07 | 5.331E-06 |
| CST1 | -0.059 | -0.091 | -0.026 | 0.0004078 | 0.0024885 |
| CRISP2 | -0.060 | -0.083 | -0.036 | 8.964E-07 | 9.777E-06 |
| KLK3 | -0.061 | -0.098 | -0.024 | 0.0011121 | 0.0059555 |
| CTSB | -0.062 | -0.084 | -0.041 | 2.369E-08 | 3.763E-07 |
| ADAMTS8 | -0.064 | -0.083 | -0.046 | 1.372E-11 | 4.312E-10 |
| AFP | -0.065 | -0.092 | -0.038 | 2.733E-06 | 2.654E-05 |
| PPY | -0.067 | -0.109 | -0.026 | 0.0015426 | 0.0079807 |
| RAB6A | -0.068 | -0.087 | -0.049 | 2.653E-12 | 1.007E-10 |
| CELA3A | -0.069 | -0.093 | -0.046 | 4.899E-09 | 9.121E-08 |
| TSHB | -0.069 | -0.1 | -0.038 | 1.223E-05 | 0.0001027 |
| UMOD | -0.071 | -0.094 | -0.049 | 3.66E-10 | 8.406E-09 |
| IGFBP1 | -0.074 | -0.128 | -0.018 | 0.0088612 | 0.0358637 |
| CELA2A | -0.077 | -0.098 | -0.055 | 1.86E-12 | 7.154E-11 |
| CA6 | -0.083 | -0.109 | -0.057 | 4.894E-10 | 1.076E-08 |
| KLK1 | -0.093 | -0.144 | -0.042 | 0.0003776 | 0.0023236 |
| OMG | -0.093 | -0.121 | -0.066 | 2.724E-11 | 7.731E-10 |
| XPNPEP2 | -0.103 | -0.143 | -0.063 | 5.398E-07 | 6.211E-06 |
| GP2 | -0.112 | -0.146 | -0.078 | 1.104E-10 | 2.796E-09 |
| BPIFA2 | -0.123 | -0.161 | -0.086 | 1.38E-10 | 3.389E-09 |

**Supplement Table 22. GO, KEGG enrichment analysis of DJD subtype-associated proteins**

| **Group** | **ID** | **Description** | **GeneRatio** | **BgRatio** | **pvalue** | **p.adjust** | **qvalue** |
| --- | --- | --- | --- | --- | --- | --- | --- |
| OA | hsa04210 | Apoptosis | 51/1146 | 60/1708 | 0.0012381 | 0.1376195 | 0.1278865 |
| OA | hsa05167 | Kaposi sarcoma-associated herpesvirus infection | 49/1146 | 58/1708 | 0.0020558 | 0.1376195 | 0.1278865 |
| OA | hsa01521 | EGFR tyrosine kinase inhibitor resistance | 32/1146 | 36/1708 | 0.0023477 | 0.1376195 | 0.1278865 |
| OA | hsa00564 | Glycerophospholipid metabolism | 15/1146 | 15/1708 | 0.0024393 | 0.1376195 | 0.1278865 |
| OA | hsa04142 | Lysosome | 43/1146 | 51/1708 | 0.0042432 | 0.1376195 | 0.1278865 |
| OA | hsa05417 | Lipid and atherosclerosis | 69/1146 | 86/1708 | 0.004253 | 0.1376195 | 0.1278865 |
| OA | hsa04610 | Complement and coagulation cascades | 49/1146 | 59/1708 | 0.0043006 | 0.1376195 | 0.1278865 |
| OA | hsa04668 | TNF signaling pathway | 46/1146 | 55/1708 | 0.0043006 | 0.1376195 | 0.1278865 |
| OA | hsa04620 | Toll-like receptor signaling pathway | 35/1146 | 41/1708 | 0.0066213 | 0.1852699 | 0.1721669 |
| OA | hsa04215 | Apoptosis - multiple species | 17/1146 | 18/1708 | 0.0072371 | 0.1852699 | 0.1721669 |
| OA | hsa04071 | Sphingolipid signaling pathway | 24/1146 | 27/1708 | 0.0088699 | 0.2064259 | 0.1918267 |
| IVDD | hsa04820 | Cytoskeleton in muscle cells | 26/286 | 79/1708 | 0.0002361 | 0.0528819 | 0.0526831 |
| IVDD | hsa00980 | Metabolism of xenobiotics by cytochrome P450 | 7/286 | 12/1708 | 0.0012715 | 0.1424024 | 0.1418671 |
| DSD | hsa04060 | Cytokine-cytokine receptor interaction | 82/470 | 189/1708 | 4.60E-07 | 0.0001122 | 0.0001108 |
| DSD | hsa04974 | Protein digestion and absorption | 19/470 | 35/1708 | 0.0006597 | 0.04523 | 0.0446837 |
| DSD | hsa04610 | Complement and coagulation cascades | 28/470 | 59/1708 | 0.0006977 | 0.04523 | 0.0446837 |
| DSD | hsa04142 | Lysosome | 25/470 | 51/1708 | 0.0007415 | 0.04523 | 0.0446837 |
| DSD | hsa04640 | Hematopoietic cell lineage | 28/470 | 60/1708 | 0.0009763 | 0.047644 | 0.0470685 |
| DSD | hsa04061 | Viral protein interaction with cytokine and cytokine receptor | 31/470 | 71/1708 | 0.0020711 | 0.0842249 | 0.0832075 |
| OA | GO:0000165 | MAPK cascade | 208/1860 | 265/2817 | 2.33E-06 | 0.0065457 | 0.0060651 |
| OA | GO:0060326 | cell chemotaxis | 121/1860 | 147/2817 | 5.42E-06 | 0.0065457 | 0.0060651 |
| OA | GO:0016477 | cell migration | 372/1860 | 500/2817 | 5.85E-06 | 0.0065457 | 0.0060651 |
| OA | GO:0002684 | positive regulation of immune system process | 256/1860 | 336/2817 | 1.16E-05 | 0.0069777 | 0.0064654 |
| OA | GO:0032101 | regulation of response to external stimulus | 280/1860 | 373/2817 | 3.46E-05 | 0.0145063 | 0.0134413 |
| OA | GO:0050776 | regulation of immune response | 226/1860 | 297/2817 | 4.79E-05 | 0.0160961 | 0.0149143 |
| OA | GO:0050865 | regulation of cell activation | 200/1860 | 261/2817 | 6.53E-05 | 0.0199182 | 0.0184558 |
| OA | GO:0002687 | positive regulation of leukocyte migration | 65/1860 | 76/2817 | 9.24E-05 | 0.0236441 | 0.0219081 |
| OA | GO:0045321 | leukocyte activation | 263/1860 | 352/2817 | 0.000112 | 0.0250727 | 0.0232318 |
| OA | GO:0009617 | response to bacterium | 184/1860 | 240/2817 | 0.0001254 | 0.0263153 | 0.0243832 |
| OA | GO:0009607 | response to biotic stimulus | 358/1860 | 491/2817 | 0.0001998 | 0.0319614 | 0.0296148 |
| OA | GO:0001775 | cell activation | 302/1860 | 410/2817 | 0.0002084 | 0.0319614 | 0.0296148 |
| OA | GO:0001944 | vasculature development | 205/1860 | 271/2817 | 0.0002095 | 0.0319614 | 0.0296148 |
| OA | GO:0007167 | enzyme-linked receptor protein signaling pathway | 244/1860 | 327/2817 | 0.0002389 | 0.0321643 | 0.0298028 |
| OA | GO:0001836 | release of cytochrome c from mitochondria | 19/1860 | 19/2817 | 0.000364 | 0.0359442 | 0.0333052 |
| OA | GO:0097194 | execution phase of apoptosis | 19/1860 | 19/2817 | 0.000364 | 0.0359442 | 0.0333052 |
| OA | GO:0006935 | chemotaxis | 194/1860 | 257/2817 | 0.0003858 | 0.0359787 | 0.0333371 |
| OA | GO:0042330 | taxis | 194/1860 | 257/2817 | 0.0003858 | 0.0359787 | 0.0333371 |
| OA | GO:0001890 | placenta development | 40/1860 | 45/2817 | 0.0004025 | 0.0365195 | 0.0338382 |
| OA | GO:0002683 | negative regulation of immune system process | 135/1860 | 174/2817 | 0.0004245 | 0.0375015 | 0.0347481 |
| OA | GO:0040011 | locomotion | 336/1860 | 463/2817 | 0.0005991 | 0.0457118 | 0.0423556 |
| OA | GO:0010821 | regulation of mitochondrion organization | 35/1860 | 39/2817 | 0.0006293 | 0.0459231 | 0.0425514 |
| OA | GO:0001525 | angiogenesis | 169/1860 | 223/2817 | 0.0006754 | 0.0482419 | 0.0446999 |
| OA | GO:0051241 | negative regulation of multicellular organismal process | 257/1860 | 349/2817 | 0.0006908 | 0.0483112 | 0.0447642 |
| DSD | GO:0051241 | negative regulation of multicellular organismal process | 135/732 | 349/2817 | 1.70E-08 | 5.57E-05 | 4.96E-05 |
| DSD | GO:0044409 | entry into host | 42/732 | 77/2817 | 6.17E-08 | 8.98E-05 | 7.99E-05 |
| DSD | GO:0046718 | viral entry into host cell | 41/732 | 75/2817 | 8.21E-08 | 8.98E-05 | 7.99E-05 |
| DSD | GO:0002683 | negative regulation of immune system process | 75/732 | 174/2817 | 3.07E-07 | 0.0002205 | 0.0001962 |
| DSD | GO:0044000 | movement in host | 42/732 | 81/2817 | 4.08E-07 | 0.0002205 | 0.0001962 |
| DSD | GO:0002687 | positive regulation of leukocyte migration | 40/732 | 76/2817 | 4.64E-07 | 0.0002205 | 0.0001962 |
| DSD | GO:0002684 | positive regulation of immune system process | 126/732 | 336/2817 | 4.70E-07 | 0.0002205 | 0.0001962 |
| DSD | GO:0009607 | response to biotic stimulus | 170/732 | 491/2817 | 1.74E-06 | 0.0007127 | 0.0006341 |
| DSD | GO:0050865 | regulation of cell activation | 100/732 | 261/2817 | 2.89E-06 | 0.0010232 | 0.0009104 |
| DSD | GO:0048771 | tissue remodeling | 35/732 | 67/2817 | 3.12E-06 | 0.0010232 | 0.0009104 |
| DSD | GO:0002819 | regulation of adaptive immune response | 40/732 | 81/2817 | 3.92E-06 | 0.0011707 | 0.0010416 |
| DSD | GO:0051701 | biological process involved in interaction with host | 42/732 | 87/2817 | 4.73E-06 | 0.0012844 | 0.0011428 |
| DSD | GO:0043207 | response to external biotic stimulus | 165/732 | 481/2817 | 5.09E-06 | 0.0012844 | 0.0011428 |
| DSD | GO:2000026 | regulation of multicellular organismal development | 147/732 | 423/2817 | 8.68E-06 | 0.0019032 | 0.0016933 |
| DSD | GO:0001775 | cell activation | 143/732 | 410/2817 | 9.50E-06 | 0.0019032 | 0.0016933 |
| DSD | GO:0048585 | negative regulation of response to stimulus | 156/732 | 455/2817 | 1.04E-05 | 0.0019032 | 0.0016933 |
| DSD | GO:0006954 | inflammatory response | 116/732 | 322/2817 | 1.47E-05 | 0.0024106 | 0.0021449 |
| DSD | GO:0032944 | regulation of mononuclear cell proliferation | 52/732 | 121/2817 | 2.48E-05 | 0.0034849 | 0.0031007 |
| DSD | GO:0045321 | leukocyte activation | 124/732 | 352/2817 | 2.49E-05 | 0.0034849 | 0.0031007 |
| DSD | GO:0098609 | cell-cell adhesion | 129/732 | 369/2817 | 2.55E-05 | 0.0034849 | 0.0031007 |
| DSD | GO:0030155 | regulation of cell adhesion | 109/732 | 303/2817 | 3.00E-05 | 0.0036474 | 0.0032452 |
| DSD | GO:0034103 | regulation of tissue remodeling | 21/732 | 36/2817 | 3.60E-05 | 0.0039401 | 0.0035057 |
| DSD | GO:0034612 | response to tumor necrosis factor | 45/732 | 102/2817 | 4.06E-05 | 0.0042945 | 0.003821 |
| DSD | GO:0002250 | adaptive immune response | 75/732 | 195/2817 | 4.95E-05 | 0.0045351 | 0.0040351 |
| DSD | GO:0052372 | modulation by symbiont of entry into host | 13/732 | 18/2817 | 5.02E-05 | 0.0045351 | 0.0040351 |
| DSD | GO:0071356 | cellular response to tumor necrosis factor | 42/732 | 94/2817 | 5.11E-05 | 0.0045351 | 0.0040351 |
| DSD | GO:0007167 | enzyme-linked receptor protein signaling pathway | 115/732 | 327/2817 | 5.67E-05 | 0.0046575 | 0.004144 |
| DSD | GO:0050870 | positive regulation of T cell activation | 45/732 | 105/2817 | 9.60E-05 | 0.0068533 | 0.0060978 |
| DSD | GO:0097530 | granulocyte migration | 38/732 | 85/2817 | 0.0001152 | 0.0077499 | 0.0068955 |
| DSD | GO:0070661 | leukocyte proliferation | 60/732 | 152/2817 | 0.0001251 | 0.0077499 | 0.0068955 |
| DSD | GO:0002706 | regulation of lymphocyte mediated immunity | 34/732 | 74/2817 | 0.0001367 | 0.0080164 | 0.0071326 |
| DSD | GO:0060326 | cell chemotaxis | 58/732 | 147/2817 | 0.0001651 | 0.008679 | 0.0077222 |
| DSD | GO:0006935 | chemotaxis | 92/732 | 257/2817 | 0.0001674 | 0.008679 | 0.0077222 |
| DSD | GO:0042330 | taxis | 92/732 | 257/2817 | 0.0001674 | 0.008679 | 0.0077222 |
| DSD | GO:0002834 | regulation of response to tumor cell | 9/732 | 11/2817 | 0.0001692 | 0.008679 | 0.0077222 |
| DSD | GO:0110148 | biomineralization | 25/732 | 50/2817 | 0.0002074 | 0.0099507 | 0.0088536 |
| DSD | GO:0008285 | negative regulation of cell population proliferation | 85/732 | 238/2817 | 0.0003317 | 0.0141412 | 0.0125821 |
| DSD | GO:0070374 | positive regulation of ERK1 and ERK2 cascade | 41/732 | 98/2817 | 0.0003673 | 0.0154592 | 0.0137549 |
| DSD | GO:0046651 | lymphocyte proliferation | 51/732 | 129/2817 | 0.0003892 | 0.0157991 | 0.0140572 |
| DSD | GO:0002252 | immune effector process | 88/732 | 249/2817 | 0.0003898 | 0.0157991 | 0.0140572 |
| DSD | GO:0045453 | bone resorption | 14/732 | 23/2817 | 0.0004132 | 0.0163431 | 0.0145413 |
| DSD | GO:0040011 | locomotion | 150/732 | 463/2817 | 0.000439 | 0.0171503 | 0.0152595 |
| DSD | GO:0002521 | leukocyte differentiation | 72/732 | 197/2817 | 0.0004498 | 0.0171696 | 0.0152767 |
| DSD | GO:0019221 | cytokine-mediated signaling pathway | 73/732 | 201/2817 | 0.0005094 | 0.0190032 | 0.0169082 |
| DSD | GO:0006959 | humoral immune response | 47/732 | 118/2817 | 0.00054 | 0.0196976 | 0.0175259 |
| DSD | GO:1902105 | regulation of leukocyte differentiation | 43/732 | 106/2817 | 0.0005887 | 0.0209384 | 0.01863 |
| DSD | GO:0010562 | positive regulation of phosphorus metabolic process | 108/732 | 320/2817 | 0.0006279 | 0.0212525 | 0.0189094 |
| DSD | GO:0045937 | positive regulation of phosphate metabolic process | 108/732 | 320/2817 | 0.0006279 | 0.0212525 | 0.0189094 |
| DSD | GO:0002449 | lymphocyte mediated immunity | 47/732 | 119/2817 | 0.0006749 | 0.022381 | 0.0199135 |
| DSD | GO:0048514 | blood vessel morphogenesis | 86/732 | 246/2817 | 0.0006842 | 0.0224619 | 0.0199855 |
| DSD | GO:0008217 | regulation of blood pressure | 23/732 | 48/2817 | 0.000806 | 0.0256896 | 0.0228574 |
| DSD | GO:0031214 | biomineral tissue development | 23/732 | 48/2817 | 0.000806 | 0.0256896 | 0.0228574 |
| DSD | GO:0009887 | animal organ morphogenesis | 84/732 | 241/2817 | 0.0008823 | 0.0274964 | 0.0244649 |
| DSD | GO:0050920 | regulation of chemotaxis | 43/732 | 108/2817 | 0.0009358 | 0.0287136 | 0.025548 |
| DSD | GO:0002418 | immune response to tumor cell | 9/732 | 13/2817 | 0.0013038 | 0.0354777 | 0.0315663 |
| DSD | GO:0002507 | tolerance induction | 9/732 | 13/2817 | 0.0013038 | 0.0354777 | 0.0315663 |
| DSD | GO:0002675 | positive regulation of acute inflammatory response | 9/732 | 13/2817 | 0.0013038 | 0.0354777 | 0.0315663 |
| DSD | GO:1903902 | positive regulation of viral life cycle | 9/732 | 13/2817 | 0.0013038 | 0.0354777 | 0.0315663 |
| DSD | GO:0045824 | negative regulation of innate immune response | 14/732 | 25/2817 | 0.0013152 | 0.0354777 | 0.0315663 |
| DSD | GO:0061061 | muscle structure development | 54/732 | 145/2817 | 0.0014357 | 0.0368229 | 0.0327632 |
| DSD | GO:0001503 | ossification | 43/732 | 110/2817 | 0.0014523 | 0.0369615 | 0.0328865 |
| DSD | GO:0002714 | positive regulation of B cell mediated immunity | 11/732 | 18/2817 | 0.0016963 | 0.0412612 | 0.0367122 |
| DSD | GO:0002891 | positive regulation of immunoglobulin mediated immune response | 11/732 | 18/2817 | 0.0016963 | 0.0412612 | 0.0367122 |
| DSD | GO:0031348 | negative regulation of defense response | 37/732 | 92/2817 | 0.0016967 | 0.0412612 | 0.0367122 |
| DSD | GO:0051146 | striated muscle cell differentiation | 28/732 | 65/2817 | 0.0018371 | 0.0424737 | 0.037791 |
| DSD | GO:0030198 | extracellular matrix organization | 47/732 | 124/2817 | 0.0019031 | 0.0427947 | 0.0380766 |
| DSD | GO:0043062 | extracellular structure organization | 47/732 | 124/2817 | 0.0019031 | 0.0427947 | 0.0380766 |
| DSD | GO:0045229 | external encapsulating structure organization | 47/732 | 124/2817 | 0.0019031 | 0.0427947 | 0.0380766 |
| DSD | GO:0001910 | regulation of leukocyte mediated cytotoxicity | 20/732 | 42/2817 | 0.0019315 | 0.0428444 | 0.0381208 |
| DSD | GO:0070371 | ERK1 and ERK2 cascade | 50/732 | 134/2817 | 0.0020418 | 0.044987 | 0.0400273 |
| DSD | GO:0030278 | regulation of ossification | 17/732 | 34/2817 | 0.002168 | 0.0464406 | 0.0413206 |
| DSD | GO:0002715 | regulation of natural killer cell mediated immunity | 14/732 | 26/2817 | 0.0021785 | 0.0464406 | 0.0413206 |
| DSD | GO:0042269 | regulation of natural killer cell mediated cytotoxicity | 14/732 | 26/2817 | 0.0021785 | 0.0464406 | 0.0413206 |
| DSD | GO:0060135 | maternal process involved in female pregnancy | 12/732 | 21/2817 | 0.0023284 | 0.0490007 | 0.0435984 |

**Supplement Table 23. Identification of ACD-associated proteins using multivariable Cox regression**

| **Proteins** | **HR** | **CI_lower** | **CI_upper** | **pvalue** | **FDR** |
| --- | --- | --- | --- | --- | --- |
| MENT | 3.224 | 2.562 | 4.057 | 1.869E-23 | 1.092E-20 |
| NEFL | 2.24 | 2.076 | 2.418 | 2.277E-95 | 3.328E-92 |
| GFAP | 2.121 | 1.995 | 2.254 | 1.06E-128 | 3.09E-125 |
| PLAUR | 1.98 | 1.701 | 2.304 | 1.161E-18 | 3.77E-16 |
| GDF15 | 1.765 | 1.63 | 1.911 | 1.747E-44 | 1.276E-41 |
| FSTL1 | 1.764 | 1.402 | 2.219 | 1.279E-06 | 2.689E-05 |
| ADM | 1.742 | 1.485 | 2.044 | 9.414E-12 | 7.644E-10 |
| GFRA1 | 1.709 | 1.473 | 1.983 | 1.608E-12 | 1.679E-10 |
| CSF1 | 1.699 | 1.449 | 1.993 | 7.358E-11 | 4.779E-09 |
| C7 | 1.698 | 1.427 | 2.02 | 2.351E-09 | 1.041E-07 |
| CD276 | 1.691 | 1.481 | 1.931 | 8.313E-15 | 1.35E-12 |
| COL18A1 | 1.686 | 1.394 | 2.038 | 6.876E-08 | 1.951E-06 |
| IL1R1 | 1.682 | 1.382 | 2.047 | 2.107E-07 | 5.309E-06 |
| LTBP2 | 1.677 | 1.46 | 1.926 | 2.791E-13 | 3.384E-11 |
| EPHA2 | 1.672 | 1.471 | 1.901 | 4.023E-15 | 7.84E-13 |
| AMBP | 1.664 | 1.339 | 2.069 | 4.575E-06 | 7.961E-05 |
| ACVRL1 | 1.659 | 1.404 | 1.96 | 2.665E-09 | 1.146E-07 |
| EDA2R | 1.652 | 1.48 | 1.845 | 3.81E-19 | 1.55E-16 |
| EBI3_IL27 | 1.646 | 1.422 | 1.904 | 2.343E-11 | 1.67E-09 |
| DCN | 1.641 | 1.356 | 1.985 | 3.566E-07 | 8.47E-06 |
| CRIM1 | 1.634 | 1.308 | 2.04 | 1.483E-05 | 0.0002054 |
| TINAGL1 | 1.621 | 1.316 | 1.997 | 5.693E-06 | 9.509E-05 |
| CD59 | 1.618 | 1.356 | 1.931 | 9.895E-08 | 2.654E-06 |
| C1QA | 1.614 | 1.315 | 1.982 | 4.746E-06 | 8.161E-05 |
| TGFBR1 | 1.613 | 1.316 | 1.979 | 4.346E-06 | 7.698E-05 |
| CLEC5A | 1.601 | 1.382 | 1.855 | 3.922E-10 | 2.205E-08 |
| LRG1 | 1.591 | 1.309 | 1.934 | 3.103E-06 | 5.788E-05 |
| WNT9A | 1.583 | 1.366 | 1.835 | 1.072E-09 | 5.274E-08 |
| SNAP25 | 1.581 | 1.417 | 1.763 | 2.108E-16 | 4.739E-14 |
| TNFRSF11B | 1.573 | 1.348 | 1.836 | 9E-09 | 3.373E-07 |
| AREG | 1.569 | 1.425 | 1.729 | 6.401E-20 | 3.118E-17 |
| CD302 | 1.563 | 1.361 | 1.794 | 2.262E-10 | 1.323E-08 |
| LIFR | 1.555 | 1.283 | 1.885 | 6.962E-06 | 0.0001124 |
| GFRA2 | 1.54 | 1.24 | 1.913 | 9.417E-05 | 0.0010045 |
| RSPO3 | 1.534 | 1.313 | 1.792 | 6.64E-08 | 1.903E-06 |
| OSMR | 1.53 | 1.216 | 1.927 | 0.0002914 | 0.0025735 |
| NCS1 | 1.529 | 1.303 | 1.794 | 1.922E-07 | 4.884E-06 |
| TNFRSF1A | 1.523 | 1.332 | 1.742 | 8.162E-10 | 4.26E-08 |
| CLEC12A | 1.515 | 1.182 | 1.942 | 0.0010326 | 0.0071812 |
| SCARB2 | 1.514 | 1.333 | 1.721 | 1.989E-10 | 1.211E-08 |
| NECTIN2 | 1.513 | 1.307 | 1.752 | 3.037E-08 | 9.544E-07 |
| PTGDS | 1.511 | 1.317 | 1.734 | 4.045E-09 | 1.62E-07 |
| IL6ST | 1.504 | 1.153 | 1.963 | 0.0026247 | 0.0146413 |
| CKAP4 | 1.488 | 1.285 | 1.722 | 1.095E-07 | 2.911E-06 |
| MERTK | 1.487 | 1.243 | 1.78 | 1.468E-05 | 0.0002053 |
| PVR | 1.487 | 1.319 | 1.677 | 9.425E-11 | 5.989E-09 |
| SHISA5 | 1.487 | 1.265 | 1.748 | 1.456E-06 | 3.018E-05 |
| CA12 | 1.486 | 1.296 | 1.705 | 1.49E-08 | 5.31E-07 |
| SPON2 | 1.484 | 1.307 | 1.685 | 1.081E-09 | 5.274E-08 |
| GGT5 | 1.481 | 1.213 | 1.808 | 0.000114 | 0.0011825 |
| TPK1 | 0.71 | 0.582 | 0.866 | 0.0007325 | 0.0054762 |
| SPOCK1 | 0.708 | 0.59 | 0.849 | 0.0001901 | 0.0018397 |
| TNFSF12 | 0.708 | 0.601 | 0.835 | 3.818E-05 | 0.0004611 |
| MCTS1 | 0.707 | 0.563 | 0.888 | 0.002858 | 0.0156397 |
| ITGB1 | 0.7 | 0.56 | 0.875 | 0.0017624 | 0.0108454 |
| ITGA2 | 0.699 | 0.597 | 0.818 | 8.4E-06 | 0.0001308 |
| SERPINI1 | 0.694 | 0.572 | 0.842 | 0.0002081 | 0.0019876 |
| AFM | 0.69 | 0.534 | 0.891 | 0.0044921 | 0.0217869 |
| KDR | 0.685 | 0.571 | 0.821 | 4.151E-05 | 0.0004893 |
| PTPRF | 0.685 | 0.585 | 0.803 | 2.919E-06 | 5.54E-05 |
| APOM | 0.679 | 0.58 | 0.796 | 1.599E-06 | 3.269E-05 |
| TNXB | 0.679 | 0.568 | 0.813 | 2.358E-05 | 0.0003063 |
| ERP44 | 0.677 | 0.564 | 0.814 | 3.099E-05 | 0.0003871 |
| ERBB2 | 0.674 | 0.557 | 0.815 | 4.896E-05 | 0.000566 |
| CDON | 0.667 | 0.56 | 0.794 | 5.477E-06 | 9.201E-05 |
| CFP | 0.666 | 0.516 | 0.861 | 0.0018829 | 0.0113245 |
| GSN | 0.664 | 0.519 | 0.849 | 0.0011022 | 0.0074925 |
| IGFBP3 | 0.66 | 0.575 | 0.758 | 3.966E-09 | 1.61E-07 |
| AHNAK | 0.652 | 0.545 | 0.778 | 2.365E-06 | 4.64E-05 |
| GHR | 0.651 | 0.544 | 0.778 | 2.459E-06 | 4.761E-05 |
| IL13RA1 | 0.65 | 0.528 | 0.8 | 4.628E-05 | 0.0005433 |
| BTD | 0.646 | 0.502 | 0.83 | 0.0006526 | 0.0049804 |
| LRRN1 | 0.646 | 0.574 | 0.727 | 3.273E-13 | 3.679E-11 |
| FN1 | 0.641 | 0.524 | 0.783 | 1.415E-05 | 0.0001989 |
| B4GAT1 | 0.64 | 0.529 | 0.773 | 3.673E-06 | 6.669E-05 |
| SELENOP | 0.639 | 0.502 | 0.813 | 0.0002719 | 0.0024388 |
| ITGAM | 0.63 | 0.543 | 0.732 | 1.545E-09 | 7.17E-08 |
| ADGRG2 | 0.628 | 0.534 | 0.74 | 2.312E-08 | 7.679E-07 |
| TTR | 0.624 | 0.504 | 0.772 | 1.48E-05 | 0.0002054 |
| ITGA11 | 0.623 | 0.546 | 0.712 | 2.735E-12 | 2.578E-10 |
| FAP | 0.615 | 0.52 | 0.727 | 1.302E-08 | 4.697E-07 |
| MEGF9 | 0.612 | 0.483 | 0.777 | 5.232E-05 | 0.0006021 |
| LRTM2 | 0.606 | 0.491 | 0.748 | 3.218E-06 | 5.916E-05 |
| NPTXR | 0.602 | 0.522 | 0.695 | 4.556E-12 | 3.917E-10 |
| MET | 0.598 | 0.476 | 0.75 | 8.435E-06 | 0.0001308 |
| LCAT | 0.595 | 0.487 | 0.727 | 3.437E-07 | 8.235E-06 |
| DPP4 | 0.594 | 0.504 | 0.699 | 4.285E-10 | 2.363E-08 |
| SERPINA5 | 0.583 | 0.482 | 0.705 | 2.689E-08 | 8.732E-07 |
| TNFSF10 | 0.579 | 0.483 | 0.694 | 3.244E-09 | 1.336E-07 |
| SERPINF2 | 0.578 | 0.389 | 0.859 | 0.0066411 | 0.0287583 |
| KLKB1 | 0.577 | 0.465 | 0.716 | 5.83E-07 | 1.311E-05 |
| PEPD | 0.574 | 0.468 | 0.704 | 9.747E-08 | 2.638E-06 |
| BCAN | 0.566 | 0.498 | 0.644 | 4.293E-18 | 1.255E-15 |
| ERBB3 | 0.564 | 0.442 | 0.721 | 4.916E-06 | 8.404E-05 |
| HPGDS | 0.557 | 0.49 | 0.633 | 4.242E-19 | 1.55E-16 |
| VGF | 0.501 | 0.42 | 0.597 | 1.225E-14 | 1.884E-12 |
| CLEC3B | 0.483 | 0.373 | 0.626 | 3.652E-08 | 1.112E-06 |
| APOE | 0.42 | 0.378 | 0.468 | 5.598E-57 | 5.455E-54 |
| EGFR | 0.329 | 0.25 | 0.433 | 2.1E-15 | 4.385E-13 |
| ITGAV | 0.308 | 0.235 | 0.404 | 1.593E-17 | 4.234E-15 |

**Abbreviations:** FDR: False discovery rate; HR: Hazard ratio; CI: Confidence interval; ACD: All-cause dementia.

**Supplement Table 24. GO, KEGG and Reactome enrichment analysis of ACD-associated proteins**

| **ID** | **ONTOLOGY** | **Description** | **pvalue** | **Count** | **p.adjust** |
| --- | --- | --- | --- | --- | --- |
| GO:0051701 | BP | biological process involved in interaction with host | 1.83E-05 | 43 | 0.0375393 |
| GO:0007167 | BP | enzyme-linked receptor protein signaling pathway | 2.26E-05 | 125 | 0.0375393 |
| GO:0044000 | BP | movement in host | 3.70E-05 | 40 | 0.0403906 |
| GO:0042060 | BP | wound healing | 4.87E-05 | 77 | 0.0403906 |
| GO:0009986 | CC | cell surface | 0.0001031 | 152 | 0.0370137 |
| GO:0030545 | MF | signaling receptor regulator activity | 0.0001014 | 108 | 0.0256216 |
| GO:0030546 | MF | signaling receptor activator activity | 0.0001534 | 103 | 0.0256216 |
| GO:0048018 | MF | receptor ligand activity | 0.0001843 | 102 | 0.0256216 |
| GO:0005035 | MF | death receptor activity | 0.0003379 | 9 | 0.035223 |
| GO:0019955 | MF | cytokine binding | 0.0005044 | 33 | 0.0420675 |
| GO:0019838 | MF | growth factor binding | 0.0008023 | 35 | 0.0428768 |
| GO:0001618 | MF | virus receptor activity | 0.0008226 | 25 | 0.0428768 |
| GO:0140272 | MF | exogenous protein binding | 0.0008226 | 25 | 0.0428768 |
| hsa04015 | KEGG | Rap1 signaling pathway | 7.66E-05 | 38 | 0.0192145 |
| hsa04060 | KEGG | Cytokine-cytokine receptor interaction | 0.000214 | 77 | 0.0268607 |
| hsa04010 | KEGG | MAPK signaling pathway | 0.0009173 | 52 | 0.0474626 |
| hsa04510 | KEGG | Focal adhesion | 0.0010868 | 35 | 0.0474626 |
| hsa04810 | KEGG | Regulation of actin cytoskeleton | 0.0010868 | 35 | 0.0474626 |
| hsa04144 | KEGG | Endocytosis | 0.0011346 | 29 | 0.0474626 |
| hsa04151 | KEGG | PI3K-Akt signaling pathway | 0.0013705 | 62 | 0.0491439 |
| hsa04014 | KEGG | Ras signaling pathway | 0.004193 | 35 | 0.1315555 |
| hsa01521 | KEGG | EGFR tyrosine kinase inhibitor resistance | 0.0063716 | 18 | 0.158031 |
| hsa04512 | KEGG | ECM-receptor interaction | 0.0065664 | 21 | 0.158031 |
| hsa04012 | KEGG | ErbB signaling pathway | 0.0069257 | 14 | 0.158031 |
| hsa05205 | KEGG | Proteoglycans in cancer | 0.0139283 | 29 | 0.2913328 |
| hsa04540 | KEGG | Gap junction | 0.016844 | 10 | 0.3222361 |
| hsa05418 | KEGG | Fluid shear stress and atherosclerosis | 0.0179733 | 23 | 0.3222361 |
| hsa05223 | KEGG | Non-small cell lung cancer | 0.0210063 | 11 | 0.336823 |
| hsa05215 | KEGG | Prostate cancer | 0.0216459 | 17 | 0.336823 |
| hsa05417 | KEGG | Lipid and atherosclerosis | 0.0228127 | 34 | 0.336823 |
| hsa03272 | KEGG | Virion - Hepatitis viruses | 0.0263792 | 8 | 0.3678435 |
| hsa05212 | KEGG | Pancreatic cancer | 0.0281033 | 13 | 0.3712595 |
| hsa04270 | KEGG | Vascular smooth muscle contraction | 0.0310383 | 14 | 0.3794494 |
| hsa00910 | KEGG | Nitrogen metabolism | 0.0330358 | 7 | 0.3794494 |
| hsa04148 | KEGG | Efferocytosis | 0.0332585 | 20 | 0.3794494 |
| hsa04064 | KEGG | NF-kappa B signaling pathway | 0.0352827 | 22 | 0.3850416 |
| hsa04020 | KEGG | Calcium signaling pathway | 0.0372833 | 25 | 0.3899213 |
| hsa04390 | KEGG | Hippo signaling pathway | 0.0444572 | 15 | 0.4252592 |
| hsa04630 | KEGG | JAK-STAT signaling pathway | 0.0455508 | 33 | 0.4252592 |
| hsa05165 | KEGG | Human papillomavirus infection | 0.045745 | 32 | 0.4252592 |
| R-HSA-9772572 | Reactome | Early SARS-CoV-2 Infection Events | 3.06E-06 | 12 | 0.0016135 |
| R-HSA-5669034 | Reactome | TNFs bind their physiological receptors | 9.08E-06 | 16 | 0.0023916 |
| R-HSA-9694614 | Reactome | Attachment and Entry | 3.18E-05 | 10 | 0.0055939 |
| R-HSA-1474244 | Reactome | Extracellular matrix organization | 0.0006929 | 56 | 0.0750798 |
| R-HSA-977225 | Reactome | Amyloid fiber formation | 0.0007123 | 14 | 0.0750798 |
| R-HSA-5668541 | Reactome | TNFR2 non-canonical NF-kB pathway | 0.0029442 | 21 | 0.2510862 |
| R-HSA-1474228 | Reactome | Degradation of the extracellular matrix | 0.0049725 | 26 | 0.2510862 |
| R-HSA-2173789 | Reactome | TGF-beta receptor signaling activates SMADs | 0.0052164 | 10 | 0.2510862 |
| R-HSA-216083 | Reactome | Integrin cell surface interactions | 0.005935 | 21 | 0.2510862 |
| R-HSA-5083635 | Reactome | Defective B3GALTL causes PpS | 0.006232 | 9 | 0.2510862 |
| R-HSA-5173214 | Reactome | O-glycosylation of TSR domain-containing proteins | 0.006232 | 9 | 0.2510862 |
| R-HSA-9694516 | Reactome | SARS-CoV-2 Infection | 0.0065488 | 22 | 0.2510862 |
| R-HSA-199418 | Reactome | Negative regulation of the PI3K/AKT network | 0.0071295 | 23 | 0.2510862 |
| R-HSA-9006936 | Reactome | Signaling by TGFB family members | 0.0072854 | 17 | 0.2510862 |
| R-HSA-1236394 | Reactome | Signaling by ERBB4 | 0.0076231 | 11 | 0.2510862 |
| R-HSA-3560782 | Reactome | Diseases associated with glycosaminoglycan metabolism | 0.0076231 | 11 | 0.2510862 |
| R-HSA-186763 | Reactome | Downstream signal transduction | 0.0084049 | 7 | 0.2605523 |
| R-HSA-2219530 | Reactome | Constitutive Signaling by Aberrant PI3K in Cancer | 0.0092962 | 19 | 0.267126 |
| R-HSA-3781865 | Reactome | Diseases of glycosylation | 0.0110577 | 21 | 0.267126 |
| R-HSA-186797 | Reactome | Signaling by PDGF | 0.0123486 | 13 | 0.267126 |
| R-HSA-6806667 | Reactome | Metabolism of fat-soluble vitamins | 0.0123486 | 13 | 0.267126 |
| R-HSA-975634 | Reactome | Retinoid metabolism and transport | 0.0123486 | 13 | 0.267126 |
| R-HSA-196854 | Reactome | Metabolism of vitamins and cofactors | 0.0137883 | 25 | 0.267126 |
| R-HSA-3560783 | Reactome | Defective B4GALT7 causes EDS, progeroid type | 0.0142835 | 8 | 0.267126 |
| R-HSA-4420332 | Reactome | Defective B3GALT6 causes EDSP2 and SEMDJL1 | 0.0142835 | 8 | 0.267126 |
| R-HSA-9634638 | Reactome | Estrogen-dependent nuclear events downstream of ESR-membrane signaling | 0.0142835 | 8 | 0.267126 |
| R-HSA-3000171 | Reactome | Non-integrin membrane-ECM interactions | 0.0145131 | 14 | 0.267126 |
| R-HSA-1280215 | Reactome | Cytokine Signaling in Immune system | 0.014616 | 94 | 0.267126 |
| R-HSA-6811558 | Reactome | PI5P, PP2A and IER3 Regulate PI3K/AKT Signaling | 0.0146995 | 21 | 0.267126 |
| R-HSA-9679506 | Reactome | SARS-CoV Infections | 0.0164132 | 32 | 0.2883244 |
| R-HSA-180292 | Reactome | GAB1 signalosome | 0.017399 | 7 | 0.29244 |
| R-HSA-194138 | Reactome | Signaling by VEGF | 0.0183062 | 16 | 0.29244 |
| R-HSA-174824 | Reactome | Plasma lipoprotein assembly, remodeling, and clearance | 0.0183122 | 13 | 0.29244 |
| R-HSA-381426 | Reactome | Regulation of Insulin-like Growth Factor (IGF) transport and uptake by Insulin-like Growth Factor Binding Proteins (IGFBPs) | 0.0195895 | 30 | 0.2994143 |
| R-HSA-9013404 | Reactome | RAC2 GTPase cycle | 0.0198852 | 9 | 0.2994143 |
| R-HSA-170834 | Reactome | Signaling by TGF-beta Receptor Complex | 0.0231483 | 12 | 0.3215853 |
| R-HSA-9009391 | Reactome | Extra-nuclear estrogen signaling | 0.0231483 | 12 | 0.3215853 |
| R-HSA-1971475 | Reactome | A tetrasaccharide linker sequence is required for GAG synthesis | 0.025019 | 8 | 0.3215853 |
| R-HSA-3000170 | Reactome | Syndecan interactions | 0.025019 | 8 | 0.3215853 |
| R-HSA-3560801 | Reactome | Defective B3GAT3 causes JDSSDHD | 0.025019 | 8 | 0.3215853 |
| R-HSA-8863795 | Reactome | Downregulation of ERBB2 signaling | 0.025019 | 8 | 0.3215853 |
| R-HSA-8957275 | Reactome | Post-translational protein phosphorylation | 0.0279619 | 26 | 0.3508547 |
| R-HSA-9013423 | Reactome | RAC3 GTPase cycle | 0.0293262 | 11 | 0.3594164 |
| R-HSA-2219528 | Reactome | PI3K/AKT Signaling in Cancer | 0.0314524 | 21 | 0.3767144 |
| R-HSA-1250196 | Reactome | SHC1 events in ERBB2 signaling | 0.0396404 | 6 | 0.4085878 |
| R-HSA-180336 | Reactome | SHC1 events in EGFR signaling | 0.0396404 | 6 | 0.4085878 |
| R-HSA-2022928 | Reactome | HS-GAG biosynthesis | 0.0396404 | 6 | 0.4085878 |
| R-HSA-5339562 | Reactome | Uptake and actions of bacterial toxins | 0.0396404 | 6 | 0.4085878 |
| R-HSA-8876198 | Reactome | RAB GEFs exchange GTP for GDP on RABs | 0.0396404 | 6 | 0.4085878 |
| R-HSA-9696270 | Reactome | RND2 GTPase cycle | 0.0396404 | 6 | 0.4085878 |
| R-HSA-2142753 | Reactome | Arachidonic acid metabolism | 0.0403161 | 8 | 0.4085878 |
| R-HSA-8963899 | Reactome | Plasma lipoprotein remodeling | 0.0403161 | 8 | 0.4085878 |
| R-HSA-8856825 | Reactome | Cargo recognition for clathrin-mediated endocytosis | 0.041384 | 15 | 0.4114974 |
| R-HSA-9709957 | Reactome | Sensory Perception | 0.0472523 | 19 | 0.4317007 |
| R-HSA-1227986 | Reactome | Signaling by ERBB2 | 0.0474741 | 9 | 0.4317007 |
| R-HSA-1592389 | Reactome | Activation of Matrix Metalloproteinases | 0.0474741 | 9 | 0.4317007 |
